# Mechanical stimulation-induced purinome priming fosters osteogenic differentiation and osteointegration of mesenchymal stem cells from the bone marrow of post-menopausal women

**DOI:** 10.1186/s13287-024-03775-4

**Published:** 2024-06-18

**Authors:** Catarina Bessa-Andrês, Rui Pinto-Cardoso, Karyna Tarasova, Ana Luísa Pereira-Gonçalves, Joana Maria Gaio-Ferreira-Castro, Liliana S. Carvalho, Maria Adelina Costa, Fátima Ferreirinha, Ana Canadas-Sousa, José Marinhas, Rolando Freitas, Rui Lemos, Adélio Vilaça, António Oliveira, Paulo Correia-de-Sá, José Bernardo Noronha-Matos

**Affiliations:** 1 Laboratório de Farmacologia e Neurobiologia, Instituto de Ciências Biomédicas Abel Salazar - Universidade do Porto (ICBAS-UP), Porto, 4050-313 Portugal; 2 Center for Drug Discovery and Innovative Medicines (MedInUP), Instituto de Ciências Biomédicas Abel Salazar - Universidade do Porto (ICBAS-UP), Porto, 4050-313 Portugal; 3 Departamento de Química, Instituto de Ciências Biomédicas Abel Salazar - Universidade do Porto (ICBAS-UP), Porto, 4050-313 Portugal; 4 Departamento de Patologia e Imunologia Molecular, Instituto de Ciências Biomédicas Abel Salazar - Universidade do Porto (ICBAS-UP), Porto, 4050-313 Portugal; 5 Serviço de Ortopedia e Traumatologia, Centro Hospitalar de Vila Nova de Gaia – Espinho, Vila Nova de Gaia, 4434-502 Portugal; 6 Serviço de Ortopedia, Centro Hospitalar Universitário de Santo António, Porto, 4099-001 Portugal

**Keywords:** Mesenchymal stem cells, Post-menopausal osteogenesis, Purinergic signalling, Mechanical stimulation, P2X7 receptor, P2Y_6_ receptor

## Abstract

**Background:**

Mechanical stimulation (MS) significantly increases the release of adenine and uracil nucleotides from bone marrow-derived mesenchymal stem cells (BM-MSCs) undergoing osteogenic differentiation. Released nucleotides acting via ionotropic P2X7 and metabotropic P2Y_6_ purinoceptors sensitive to ATP and UDP, respectively, control the osteogenic commitment of BM-MSCs and, thus, bone growth and remodelling. Yet, this mechanism is impaired in post-menopausal (Pm)-derived BM-MSCs, mostly because NTPDase3 overexpression decreases the extracellular accumulation of nucleotides below the levels required to activate plasma membrane-bound P2 purinoceptors. This prompted us to investigate whether in vitro MS of BM-MSCs from Pm women could rehabilitate their osteogenic commitment and whether xenotransplantation of MS purinome-primed Pm cells promote repair of critical bone defects in an in vivo animal model.

**Methods:**

BM-MSCs were harvested from the neck of femora of Pm women (70 ± 3 years old) undergoing total hip replacement. The cells grew, for 35 days, in an osteogenic-inducing medium either submitted (SS) or not (CTR) to MS (90 r.p.m. for 30 min) twice a week. Increases in alkaline phosphatase activity and in the amount of osteogenic transcription factors, osterix and osteopontin, denoted osteogenic cells differentiation, while bone nodules formation was ascertain by the alizarin red-staining assay. The luciferin-luciferase bioluminescence assay was used to quantify extracellular ATP. The kinetics of the extracellular ATP (100 µM) and UDP (100 µM) catabolism was assessed by HPLC. The density of P2Y_6_ and P2X7 purinoceptors in the cells was assessed by immunofluorescence confocal microscopy. MS-stimulated BM-MSCs from Pm women were xenotransplanted into critical bone defects drilled in the great trochanter of femora of one-year female Wistar rats; bone repair was assessed by histological analysis 10 days after xenotransplantation.

**Results:**

MS-stimulated Pm BM-MSCs in culture (i) release 1.6-fold higher ATP amounts, (ii) overexpress P2X7 and P2Y_6_ purinoceptors, (iii) exhibit higher alkaline phosphatase activity and overexpress the osteogenic transcription factors, osterix and osteopontin, and (iv) form larger bone nodules, than CTR cells. Selective blockage of P2X7 and P2Y_6_ purinoceptors with A438079 (3 µM) and MRS 2578 (0.1 µM), respectively, prevented the osteogenic commitment of cultured Pm BM-MSCs. Xenotransplanted MS purinome-primed Pm BM-MSCs accelerated the repair of critical bone defects in the in vivo rat model.

**Conclusions:**

Data suggest that in vitro MS restores the purinergic cell-to-cell communication fostering the osteogenic differentiation and osteointegration of BM-MSCs from Pm women, a strategy that may be used in bone regeneration and repair tactics.

**Supplementary Information:**

The online version contains supplementary material available at 10.1186/s13287-024-03775-4.

## Introduction

Age-related bone diseases are a major public health burden that affects hundreds of millions of people worldwide. Osteoporosis is characterized by the loss of bone mass and skeletal impairment, which subsequently decreases bone resistance and increases the risk of hip, spine and wrist fractures [1]. Osteoporotic fractures occur at least once in each three women and roughly in one fifth of men over the age of 50 [2]. The standard medical treatment involves promoting osteoblasts activity (anabolic strategies) and/or inhibiting bone resorption (antiresorptive strategies), using hormones or chemical compounds to slow down the bone turnover rate [3]. The pharmacological strategies display several disadvantages in the long-term use [4]. Over the past few decades, new approaches have emerged with the advent of regenerative medicine, namely by the use of mesenchymal stem cells (MSCs), which are multipotent cells [5] displaying self-renewing ability [6] and exhibiting immunosuppressive properties [7].

Although the bone fracture risk in post-menopausal (Pm) women might result from excessive bone resorption, our research group demonstrated that osteoprogenitor bone marrow-derived MSCs (BM-MSCs) retain their proliferative potential throughout life, but their ability to differentiate into bone-forming osteoblasts is severely compromised [8–10]. Among many other local factors, adenine and uracil nucleotides, namely ATP and UDP, are paramount to regulate the osteogenic commitment of BM-MSCs, via the activation of ionotropic P2X7 and metabotropic P2Y_6_ purinoceptors, respectively (for a review, see [11]). Interestingly, we found that the osteogenic activity of these two purinoceptors is diminished in BM-MSCs from Pm women compared to younger females and aged-matched men ([8–10]; reviewed in [11]). Experimental data using Pm BM-MSCs undergoing osteogenic differentiation in culture showed that rehabilitation of the osteogenic commitment of these cells may be achived through activation of P2X7 and P2Y_6_ purinoceptors using enzymatically stable ATP and UDP analogues, but not when the cells were treated with the native compounds. These findings, together with the discovery that Pm MSCs overexpress the nucleotide hydrolysing enzyme, NTPDase3 [10], explain the failure of endogenous adenine and uracil nucleotides to reach high enough extracellular concentrations to activate membrane-bound purinoceptors, which is needed to trigger the osteogenic differentiation of these cells [12, 13].

The skeleton is particularly sensitive to mechanical loading in order to guide resident cell populations towards bone remodelling and repair [14]. Dynamic mechanical loading increases bone density and strength by promoting osteoblast proliferation, differentiation and matrix production [15]. When submitted to mechanical stress, BM-MSCs and differentiated osteoblasts release huge amounts of adenine and uracil nucleotides [16]. Interestingly, the expression and function of the P2X7 receptor is also highly increased upon mechanical stimulation of the cells; in this context, shockwaves enhance the osteogenic differentiation of human MSCs through the release of ATP and subsequent P2X7 receptor activation [17].

Considering the aforementioned findings, in this study we now tested a novel “non-drug” approach to investigate (1) whether in vitro MS of BM-MSCs harvested from Pm women could rehabilitate their osteogenic commitment via P2X7 and P2Y_6_ purinoceptors activation, and (2) if xenotransplantation of MS purinome-primed Pm BM-MSCs could promote repair of critical bone defects in an in vivo animal model. The possibility to use this drug-free “purinome”-priming technique in tissue remodelling and bone repair tactics will be discussed in the context of tissue engineering for bone loss and fracture mal-union situations.

## Materials and methods

### Reagents and antibodies

ATP, quinacrine mustard dihydrochloride, 3,4-dihydroxy-9,10-dioxo-2- anthracenesulfonic acid sodium salt (Alizarin red S), 3-[4,5-dimethylthiazol-2-yl]-2,5- diphenyltetrasodium bromide (MTT), phosphate-buffered saline solution (PBS), *p*-nitrophenyl phosphate (PNP) and cell culture reagents were obtained from Sigma-Aldrich (St. Louis, MO, USA; RRID: SCR_008988). 3-[[5-(2,3-dichlorophenyl)-1 H-tetrazol-1-yl]methyl]pyridine hydrochloride (A438079) and N, N’’-1,4-butanediylbis[N’-(3- isothiocyanatophenyl) thiourea (MRS 2578) were obtained from Tocris Bioscience (Bristol, UK; RRID: SCR_003689). Bovine Collagen I (A10644-01) was supplied by Thermo Fisher Scientific (Grand Island, NY, USA; RRID: SCR_008452). All primary anti-human and secondary conjugated antibodies used in this study have been previously validated [8, 9]. Anti-human P2X7 (anti-rabbit, Cat. No. APR-008, RRID: AB_2040065) and P2Y_6_ (anti-rabbit, Cat. No. APR-011, RRID: AB_2040082) were purchased from Alomone (Jerusalem, Israel); anti-human Osterix (anti-rabbit, Cat No. ab22552, RRID: AB_2194492), anti-human β-Tubulin (anti-rabbit, Cat. No. ab6046, RRID: AB_2210370) and horseradish-peroxidase-conjugated secondary antibodies (anti-rabbit, Cat. No. ab7083, RRID: AB_955416 and anti-mouse, Cat No. ab6820, RRID: AB_955438) were purchased from Abcam (Cambridge, UK; RRID: SCR_012931); anti-human Osterix (anti-mouse, Cat. No. sc-393,325, RRID: AB_2895257) and anti-human Osteopontin (anti-mouse, Cat. No. sc-21,742, RRID: AB_2194997) were purchased from Santa Cruz Biotechnology (Santa Cruz, CA, USA; RRID: SCR_008987); secondary antibody Alexa Fluor 488-labelled (anti-rabbit, Cat. No. A21206, RRID: AB_2535792) was supplied by Molecular Probes (Invitrogen, Carlsbad, CA, USA; RRID: SCR_013318).

### Cell culture conditions and phenotypic characterization of Pm BM-MSCs

Bone marrow samples were obtained from the neck of the femur of thirteen Pm women (70 ± 3 years old) undergoing total hip arthroplasty to resolve non-inflammatory degenerative osteoarthrosis. Handling of bone marrow samples and culture of adherent cells was performed until near confluence for 10–15 days, as previously described [8–10, 18]. In brief, bone marrow samples were placed immediately in fresh-frozen α-minimal essential medium (α-MEM) supplemented with 10% fetal bovine serum (FBS), 100 U/ml penicillin, 100 µg/ml streptomycin, and 2.5 µg/ml amphotericin B (standard culture medium) and transported to the laboratory on the day or following day of surgery. Bone marrow cells were dispersed on plastic dishes by repeated gently pippeting, cultured in α-MEM-based standard culture medium and incubated at 37 °C in a humidified atmosphere of 95% air and 5% CO_2_. Non-adherent cells were removed after 5 days. From this time point onwards, the culture medium of adherent cells was changed twice a week. To avoid the influence of in vitro cells senescence and phenotypic modifications, we used only first subcultures. BM-MSCs were plated at 2.5 × 10^4^ cells/mL density and were allowed to grow for 35 days in α-MEM-based standard culture medium (see the composition above) supplemented with 50 µg/mL ascorbic acid, 10 mM β-glycerophosphate and 10 nM dexamethasone to promote the osteogenic differentiation. The experimental protocols described herein are individualized, i.e. no pooled samples obtained from different individuals have been used in any circumstance.

The phenotypic characterization of the cells (first subculture) was performed previously by flow cytometry [8]. These cells exhibited positive immunoreactivity against CD105 (SH2), CD29 (integrin ß1) and CD117 (tyrosine-protein kinase Kit), which have been identified as surface markers of bone marrow-derived MSCs [19, 20]. Conversely, the cells were negative for haematopoietic surface markers, like CD14 and CD45, which have been extensively used as a good argument to distinguish bone marrow haematopoietic cells from MSCs [20, 21]. Thus, first passage plastic-adherent human bone marrow cells obtained under the present experimental conditions are highly enriched in multipotent MSCs [8].

### Mechanical stimulation (MS) of Pm BM-MSCs

Pm BM-MSCs were cultured as described above for 35 days in an osteogenic inducing medium either in the absence or presence of test drugs, namely A438079 (3 µM, P2X7 receptor antagonist) and MRS 2578 (0.1 µM, P2Y_6_ receptor antagonist), which were added to the culture medium on day 1 (first subculture). MS of the cells consisted of a previously validated shear stress (SS) protocol (see e.g. [22, 23]) and used with minor modifications; the see-saw system used in previous works and in this study produces low-magnitude fluid-SS in standard culture dishes/plates. After allowing cells adhesion to the bottom of the culture dishes for 4 days, SS cells were mechanically-stimulated twice a week using a microplate shaker placed inside an incubator (980121EU-VWR, 90 r.p.m. for 30 min, at 37ºC). Culture media changes were made twice a week, always taking care to allow a 24 h-recovery time before submitting the cells to SS protocols. Cells proliferation and differentiation were assessed at culture days 7, 14 and 21; bone nodules formation were evaluated at culture day 35. Protein was collected from the cultures at day 21 for Western blot analysis of osterix (OSX) and osteopontin (OPN), as previously described [9, 10]. Intra- and extracellular ATP amounts were evaluated using the quinacrine staining assay and the luciferin-luciferase ATP bioluminescence assay, respectively, at culture days 7 and 21. The density of P2Y_6_ and P2X7 purinoceptors in the cells was assessed by immunofluorescence confocal microscopy at culture days 7 and 21. The kinetics of the extracellular breakdown of ATP/UDP and metabolites formation was measured by HPLC at days 7 and 21. All these assays were repeated in non-stimulated (CTR) cultures for adequate comparisons (see below).

### Viability/proliferation and osteogenic differentiation of Pm BM-MSCs

Cell viability/proliferation was evaluated by the MTT assay [8–10, 18]. Data from this assay correlates positively with the results measuring cell proliferation from total DNA quantification per culture well [cf. in [8]].

The osteogenic differentiation of BM-MSCs was inferred as increases in the alkaline phosphatase (ALP) activity and in the expression of osteogenic transcription factors, osterix and osteopontin. The ALP activity was determined in cell lysates by colorimetric determination of *p*-nitrophenyl phosphate (PNP) hydrolysis, as previously described [8–10, 18]; obtained values were expressed in nanomole of PNP per min normalized by the MTT value (nmol min^− 1^ MTT^− 1^).

The total amounts of osterix (OSX) and osteopontin (OPN) proteins were determined by Western blot analysis at culture day 21, as previously described [9, 10]. Equal protein amounts (25 µg) were loaded into sodium dodecyl sulphate-polyacrylamide gel electrophoresis (SDS-PAGE) (12%) gels and transferred onto a polyvinylidene fluoride membrane using a Mini-Protean Tetra Cell coupled to a Mini-Trans-Blot module (Bio-Rad, Hercules, CA, USA; RRID: SCR_008426). Blocked membranes were incubated with anti-human primary antibodies: anti-OSX (1:1000, anti-rabbit, Cat No. ab22552, RRID: AB_2194492 or 1:200, anti-mouse, Cat. No. sc-393,325, RRID: AB_2895257) and anti-OPN (1:400, anti-mouse, Cat. No. sc-21,742, RRID: AB_2194997). Anti-β-tubulin (anti-rabbit, Cat. No. ab6046, RRID: AB_2210370) was used for normalization purposes (i.e., OSX/β-Tubulin and OPN/β-Tubulin). The peroxidase detection system (1.25 mM luminol; 0.2 mM coumaric acid; 0.1 M Tris, pH 8.5; and 0.032% hydrogen peroxide) was used for visualization of the immunoreactivity using the horseradish-peroxidase-conjugated secondary antibodies (1:70000, anti-rabbit, Cat. No. ab7083, RRID: AB_955416 and anti-mouse, Cat No. ab6820, RRID: AB_955438). Gels were analysed using a gel blot imaging system (ChemiDoc MP, RRID: SCR_019037; Bio-Rad, Hercules, CA, USA; RRID: SCR_008426).

Calcium deposition in mineralized nodules was revealed by the Alizarin Red staining and photographed using an optic microscope (Olympus CKX41, RRID: SCR_023725; Tokyo, Japan; RRID: SCR_017564) equipped with a digital camera (Olympus SC30, Tokyo, Japan; RRID: SCR_017564), running an image acquisition software (Olympus Analysis GetIT 5.1, Tokyo, Japan; RRID: SCR_017564), at culture day 35. Calibrated images were exported to Image J 1.37c software (RRID: SCR_003070; NIH, Bethesda, MD, USA) for quantification of the total bone-nodule areas [9, 10].

### Immunofluorescence staining and confocal microscopy observation of Pm BM-MSCs

Pm BM-MSCs were allowed to grow in glass chamber slides for 7 or 21 days. Paraformaldehyde fixed cells were incubated in the dark for 2 h with the following primary antibodies: rabbit anti-human P2X7 (1:75, Cat. No. APR-008, RRID: AB_2040065) and rabbit anti-human P2Y_6_ (1:75, Cat. No. APR-011, RRID: AB_2040082). Alexa Fluor 488 (1:1500, anti-rabbit, Cat. No. A21206, RRID: AB_2535792) was applied as secondary antibody for 1 h in the dark. The VectaShield mounting medium with DAPI was used to mount the glass slides, which were then stored at 4°C until visualization. Observation of the slides was made using a laser-scanning spectral confocal microscope (Olympus FV1000, RRID: SCR_020337; Tokyo, Japan; RRID: SCR_017564) built on an IX81SF-3 inverted motorized microscope with four laser lines controlled by an AOTF laser combiner. Both multi-argon laser and diode laser 405 lines, filtered by barrier filters Ion Coating for OLYMPUS UIS-2 optics, through a UPLSAPO40xOl / NA 0.5–1.0 WD 0.12 mm objective lens (Olympus, Tokyo, Japan; RRID: SCR_017564), were used to acquire images unless otherwise stated. The Fluoview FV1000 Advanced Software (4.0.3.4 version, RRID: SCR_014215; Olympus, Tokyo, Japan; RRID: SCR_017564) was used to analyse data and to control image acquisition parameters, which were set to one-way XY repeat scanning mode at 12.5 s/pixel speed with the pinhole set to 250 μm at an image resolution of 640 × 640 pixel (317.583 × 317.583 μm given that 1 pixel = 0.497 μm). Acquired micrographs were stored in the Olympus Multi TIFF format (Tokyo, Japan; RRID: SCR_017564) [8–10, 18]. For comparison purposes, confocal microscope settings and image acquisition parameters were kept unaltered throughout parallel documentation procedures. Negative controls were made in the absence of the primary antibodies or by replacing them by pre-immune sera. Unspecific fluorescence was not detected under these circumstances (data not shown). Five microscopic fields (area of approx. 93,000 µm^2^ each) were photographed per well. Standardization of XY image coordinates was as follows: the first image was taken at the centre of the well (X = 0; Y = 0) and the next four images were obtained sequentially from each corner of a hypothetical square enclosed in a circumference of 0.275 cm radius. The obtained five independent images were exported to Image J 1.37c software (RRID: SCR_003070; NIH, Bethesda, MD, USA) for quantification analysis. Regions of interest (ROIs) outlining complete individual cells were done manually and the average intensity of the pixels inside each cell was calculated per micrograph. The background fluorescence estimated from outlined ROIs drawn without transecting any cell was subtracted from all monitored ROIs. The computed analysis of the five individual images was expressed as the average fluorescence intensity (arbitrary units, a.u.) for each experimental condition. Shown in the figures are typical immunofluorescence images for each experimental condition, taken from a single representative microscopic field without juxtaposition. When necessary, software adjustments were applied to the entire image [10].

### Quinacrine-stained intracellular ATP deposits and extracellular of ATP bioluminescence

We used quinacrine fluorescence staining (ex: 476 nm / em: 500–540 nm) to visualize ATP intracellular stores. To this end, the cells were allowed to grow for 7 or 21 days in an osteogenic-inducible medium. After removing the incubation medium, the cells were washed three times with phosphate-buffered saline (PBS, 1x) and subsequently incubated for 1 h with quinacrine (30 µM), at 37ºC [24]. Images were acquired using an epifluorescence microscope equipped with a XBO 75 W Xenon arc lamp (Achroplan; Zeiss, Oberkochen, Germany; RRID: SCR_023607). The light path included ET460/30 x excitation / ET520/40 m emission filters (Chroma Technology Corp, Bellows Falls, VT, USA) and a LUMPLFLN40XW/0.80NA/3.3WD water dipping objective lens (Olympus, Tokyo, Japan; RRID: SCR_017564). A high-resolution cooled CCD camera (CoolSnap HQ, Roper Scientific Photometrics, Tucson, AZ, USA) connected to a computer running a digital image acquisition software (MetaFluor 6.3, RRID: SCR_014294; Molecular Devices Inc., Sunnyvale, CA, USA) was used to record images in the TIFF format. The CCD exposure time was set to 100 ms, binning was adjusted to 2 and gain to 1.

Extracellular ATP levels were quantified using the luciferin-luciferase ATP bioluminescence assay kit HS II (Roche Applied Science, Indianapolis, Indiana, USA) in a multi-detection microplate reader (Synergy HT, RRID: SCR_020536; BioTek Instruments, Vermont, USA), as described elsewhere [24]. Briefly, the cells were seeded onto 96-well microplates, at a density of 2.5 × 10^4^ cells/mL, for 7 or 21 days (4–8 replicas were performed per individual experiment). At the beginning of the experiment, the cells were washed twice with a Tyrode’s solution. The cells where then incubated with a fresh Tyrode’s solution for 30 min, at 37ºC. Then, the incubation fluid was removed and aliquots were snap-frozen in liquid nitrogen. Before adding the luciferin-luciferase mixture, the collected samples were defrosted until 25ºC according to the manufacturer’s instructions. Sample bioluminescence was compared to external high-purity ATP standards; these were made daily within the same concentration range; all samples were analysed in duplicates. The remaining incubation medium was used to quantify the lactate dehydrogenase (LDH, EC 1.1.1.27) activity [25] to evaluate cell integrity during the experimental period (see [24]). The LDH activity was negligible (between 0.071 and 0.12 mU/mL) in all measured samples indicating the integrity of the cells during the experimental period.

### Enzymatic kinetic experiments and HPLC analysis of extracellular nucleotides and its metabolites

The kinetics of the extracellular catabolism of ATP and UDP in Pm BM-MSCs cultures submitted or not to mechanical stimulation was evaluated on days 7 and 21 [8, 18]. After a 30-min equilibration period, the cells were incubated, at 37ºC, with gassed (95% O_2_ plus 5% CO_2_) Tyrode’s solution (137 mM NaCl, 2.7 mM KCl, 1.8 mM CaCl_2_, 1 mM MgCl_2_, 0.4 mM NaH_2_PO_4_, 11.9 mM NaHCO_3_, and 11.2 mM glucose, pH 7.4) supplemented with 100 µM ATP or UDP (zero time). Samples (75 µl) were collected from each well at different times up to 30 min for HPLC analysis (LaChrom Elite, Merck, Frankfurt, Germany) of the variation of substrate disappearance and products formation; 20-µl injection volumes were used for the analysis. The concentrations of the substrates and their respective metabolites were plotted as a function of time (progress curves). The following parameters were analysed for each progress curve: half-life time (t_1/2_) of the initial substrate, time of appearance of the different concentrations of the products, the concentration of the substrate or any product remaining at the end of the experiment. The spontaneous degradation of ATP and UDP, at 37ºC, was negligible over a period of 30-min in the absence of the cells. At the end of the experiments, the remaining incubation medium was collected and used to measure the lactate dehydrogenase (LDH, EC 1.1.1.27) activity [25]. The negligible activity of LDH in the samples collected at the end of the experiments is an indication of the integrity of the cells during the experimental procedure.

### Collagen-I encapsulation of cultured Pm BM-MSCs for xenotransplantation

For xenotransplantation, Pm BM-MSCs were cultured as described above and submitted (or not) to mechanical stimulation either in the absence or presence of selective P2X7 and P2Y_6_ receptor antagonists (A438079 3 µM and MRS 2578 0.1 µM, respectively). After allowing Pm BM-MSCs to grow in culture for day 21, they were detached and encapsulated into collagen I matrix (A10644-01, Thermo Fischer Scientific, NY, USA; RRID: SCR_008452) at a density of approximately 1 × 10^6^ cells/mL. For encapsulation, the cells were pelleted and re-suspended into sterile 15-mL Falcon tubes containing 30-µL collagen I scaffolds, which were kept for 30–40 min at 37 °C in a humidified atmosphere under 95% air and 5% CO_2_. After adding 200-µL of the osteogenic culture medium to each tube, the encapsulated cells were maintained in culture for 24 h before xenotransplantation [26].

### Critical bone defect repair using an “in vivo” animal model

Twenty female Wistar rats (*Rattus norvegicus*; Cat. No. 13,508,588, RRID: RGD_13508588; Charles River, Barcelona, Spain; RRID: SCR_003792) of about one-year old weighting 230–360 g were housed in groups of three to four animals inside ventilated Double Decker (38 cm high) cages with enriched environment and access to food and water *ad libitum*. The room temperature was kept constant (21 °C) and a regular light (07:30–19:30 h)–dark (19:30–07:30 h) cycle was imposed. The animals were acclimatized to these conditions for at least 10 days before their assignment to the experimental groups. Critical bone defects were made under general anaesthesia using 75 mg/kg ketamine (Imalgene 100 mg/mL, Boehringer Ingelheim, Germany; RRID: SCR_004791) plus 0.5 mg/kg medetomidine (Domtor 1 mg/mL, Ecuphar, Portugal) intraperitoneally. After achieving deep anaesthesia (absence of reflexes), the hind limbs were shaved and the skin disinfected. The rats were then positioned in lateral decubitus and a small incision was made in the skin below the greater trochanter of femora to expose the insertion of the rectus femoris and vastus lateralis muscles. The lateral surface of the femoral diaphysis was exposed by blunt debridement to create a critical bone defect (hole of 2.1 mm diameter) in the greater trochanter oriented towards the lesser trochanter using an electric drill (OmniDrill35, WPI, UK; RRID: SCR_008593) (see a schematic representation in Fig. 7). Drilling was made at slow speed with continuous irrigation using physiological saline to avoid heating and damage of the cells surrounding bone defects. After removing bone debris, the defect was loaded with encapsulated Pm BM-MSCs prepared as described above. All the cells used in the present study underwent osteogenic differentiation for 21 days, either in the absence (CTR group) or in presence of mechanical stimulation (SS group). The mechanically stimulated Pm BM-MSCs were subdivided into 3 groups comprising cells cultured in the absence (SS group) and in the presence of selective P2X7 (SS + A438079, 3 µM) and P2Y_6_ (SS + MRS 2578, 0.1 µM) receptor antagonists; at least 4 animals were used per experimental group and xenotransplantation of Pm BM-MSCs groups was made blindly by only one operator. Contralateral critical bone defects either empty or filled with collagen I (no added cells) scaffolds were used as controls. Bone defects were then covered with bone wax and the wound was closed by suturing soft tissue plans. After surgery, reversal of anaesthesia was achieved using 1 mg/kg IM atipamezole hydrochloride (Antisedan 5 mg/mL, Ecuphar, Portugal). Immediately after surgery and in the next following days analgesia was warranted with tramadol 10 mg/kg IM (tramadol 100 mg/2mL, Labesfal, Portugal). The follow-up recovery was monitored for 10 days after which the rats were sacrificed by decapitation followed by exsanguination and femora removed for histological analysis [26, 27].

### Histological staining and analysis

After sacrifice of the animals, both femora were removed and fixed in 10% buffered formalin for at least 72 h for histological analysis, as described previously [26, 27]. The bones were then decalcified in fresh Shandon TBD-1™ Decalcifier (Thermo Fischer Scientific, NY, USA; RRID: SCR_008452) for 48 h and, then, sectioned through the middle line after gross examination. The tissue samples were routinely processed for histological analysis. Paraffin-embedded serial sections of 4-µm thick were stained with hematoxylin and eosin (HE; general overview) and Masson’s trichrome. The latter staining was used to highlight collagen fibres allowing the distinction of the four overlapping stages of secondary bone repair [28], namely the initial inflammatory response, the soft callus formation, the hard callus formation, and the bone-remodelling phase. The NanoZoomer 2.0HT (Hamamatsu Photonics K.K., Japan; RRID: SCR_017105) was used to visualize and scan the histological glass slides, which were then converted into high-quality/high-resolution digital images using the SlideViewer 2.7 software (RRID: SCR_017654; 3DHISTECH, Budapest, Hungary). This software was also used to manually outline and quantify the following histological parameters: the total area of the bone defect, the area filled by Pm BM-MSCs (initial stage of bone regeneration characterized by MSCs recruitment), the endochondral ossification area (second stage of bone regeneration through soft callus formation), and the woven bone area (third stage of bone regeneration by hard callus formation). Two independent observers made histological analysis blindly.

### Presentation of data and statistical analysis

Data are expressed as scatter dot plot (with mean ± S.E.M.) or as *Box and Whiskers* (Min to Max) from an *n* number of individuals. No predetermined sample size calculation was performed. Due to restricted access to similar human samples and a limited pool of initial cell density, we were unable to perform all the indicated assays in all collected human samples. Normality tests included D’Agostino & Pearson and Shapiro-Wilk, depending on sample size. According to normality test results, statistical analyses included parametric (two-way ANOVA with Tukey’s test for multiple comparisons and ordinary one-way ANOVA with Fisher’s LSD test) or non-parametric tests (two-tailed Mann Whitney, Kruskal-Wallis with Dunn’s multiple comparison or uncorrected Dunn’s test and Wilcoxon matched-pairs signed rank test), with a confidence level of 0.05 (95% confidence interval). Values of *P* < 0.05 were considered to represent significant differences. Data analysis was performed using the Prism 10.0.2 TM software (RRID: SCR_002798; GraphPad Software, CA, United States).

## Results

### Mechanical stimulation (SS) increases the P2X7 and P2Y_6_ receptors immunoreactivity in Pm BM- MSCs

**Fig. 1:**
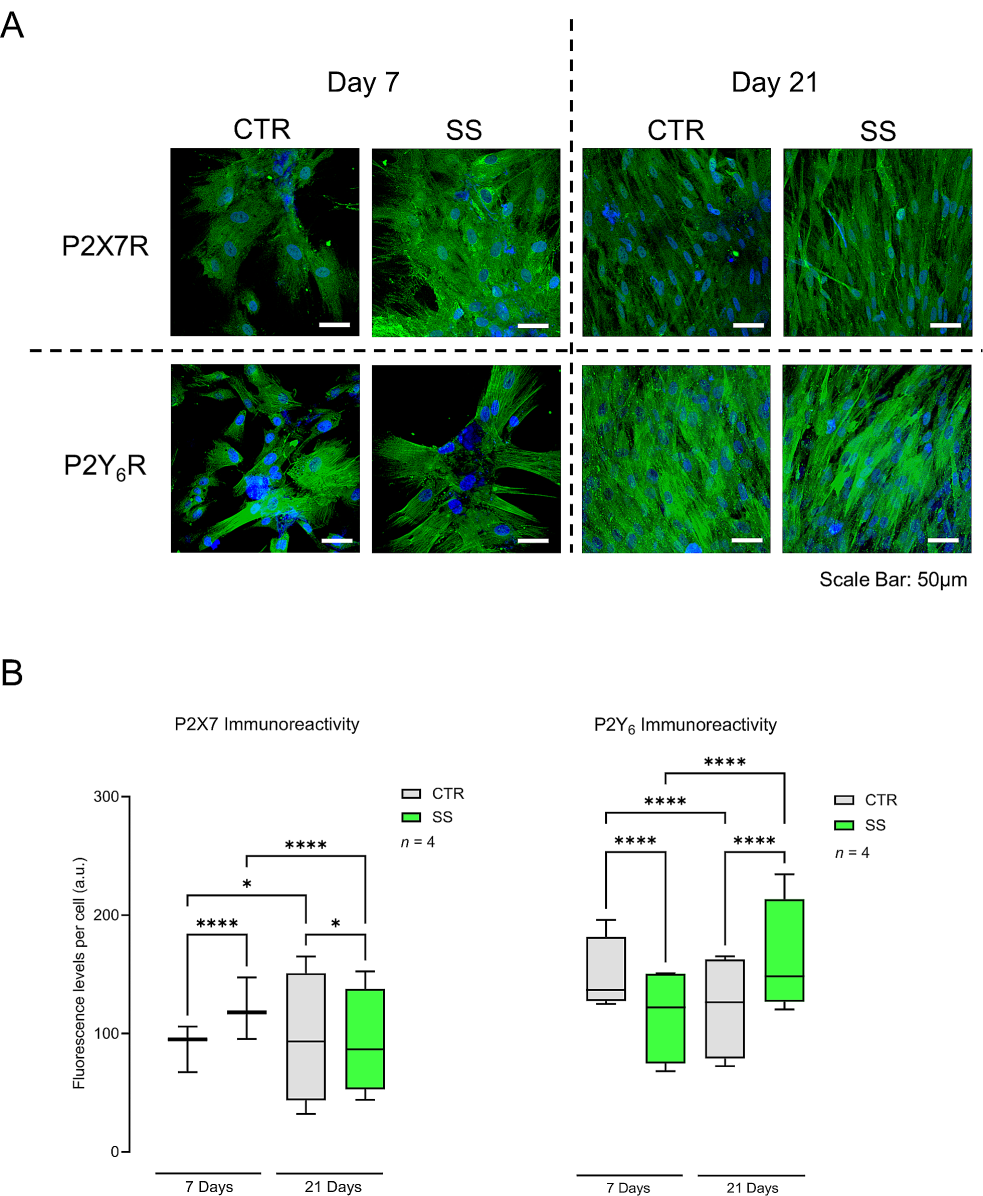
Effect of mechanical stimulation (SS) on the density of the P2X7 and P2Y_6_ receptors immunoreactivity in cultured BM-MSCs from Pm women. **Panel A** presents representative immunofluorescence confocal micrographs of BM-MSCs from a Pm woman (69 years old) grown for 7 and 21 days in an osteogenic-inducing medium stained against P2X7 and P2Y_6_ receptors (green). Blue dots represent nuclei stained with DAPI. Scale bar is 50 μm. In **panel B**, ordinates represent the fluorescence intensity per cell (arbitrary units, a.u.) of the indicated immunotarget as a function of the number of days in culture (day 7 and 21). Boxes and whiskers represent pooled data from a total of 125–317 cells analysed from 4 Pm women (75 ± 2 years old). ^*^P < 0.05 and ^****^P < 0.0001 (non-parametric Kruskal-Wallis test with Dunn’s multiple comparison test) represent significant differences

At culture day 7, the mechanically stimulated BM-MSCs isolated from Pm women (SS) overexpress the P2X7 receptor immunoreactivity compared to non-stimulated cells (CTR), but this difference was attenuated when the cells were allowed to grow in culture for 21 days (Fig. 1, left hand-side panels). Conversely, mechanically-stimulated Pm BM-MSCs (SS) exhibit a higher P2Y_6_ receptor density compared to resting cells (CTR) when the cells were allowed to grow for 21 days in culture, but not at early (7-day) culture stages (Fig. 1, right hand-side panels).

### Mechanical stimulation (SS) fosters the intracellular accumulation and outflow of ATP by Pm BM-MSCs undergoing osteogenic differentiation

**Fig. 2:**
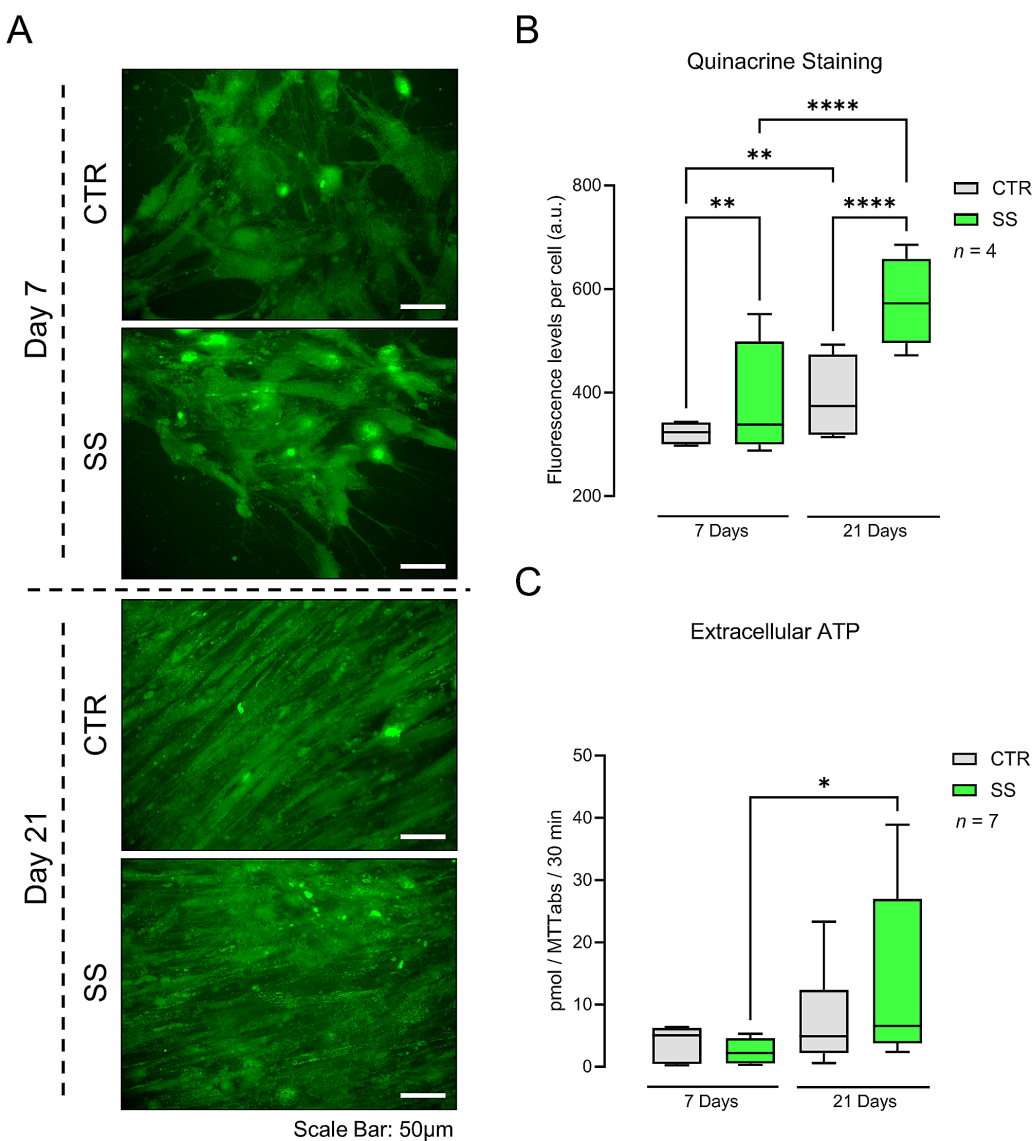
Mechanical stimulation (SS) fosters the intracellular accumulation and the outflow of ATP from Pm BM-MSCs undergoing osteogenic differentiation. Pm BM-MSCs grown in an osteoblast-inducing medium for 7 and 21 days either in the absence or in the presence of shear-stress mechanical stimulation. **Panel A**, shows the intracellular ATP accumulation in fluorescently-stained quinacrine granules imaged from Pm BM-MSCs submitted (SS) or not (CTR) to mechanical stimulation. These are representative fluorescent micrographs from BM-MSCs obtained from a 69 years-old woman. Scale bar is 50 μm. In **panel B**, ordinates represent the fluorescence intensity per cell (arbitrary units, a.u.) as a function of the number of days in culture (day 7 and 21). Boxes and whiskers represent pooled data from a total of 173–410 cells analysed from 5 Pm women (73 ± 2 years old). ^**^P < 0.01 and ^****^P < 0.0001 (non-parametric Kruskal-Wallis test with Dunn’s multiple comparison test) represent significant differences. Data in **Panel C** corresponds to the amount of ATP in the extracellular fluid obtained after 30-min incubation of Pm BM-MSCs with PBS 1x, measured using the luciferin-luciferase bioluminescence assay (see Materials and Methods). Ordinates represent ATP levels (in pmol) measured in 30-min samples corrected for cells growth/viability (MTT values) in the same well. Boxes and whiskers represent pooled data from 7 Pm women (74 ± 2 years old); four to eight replicates were performed per individual. ^*^P < 0.05 (non-parametric Kruskal-Wallis test with Dunn’s multiple comparison test) represent significant differences

Mechanically stimulated (SS) BM-MSCs isolated from Pm women accumulate significantly (*P* < 0.05) higher ATP amounts of fluorescently stained quinacrine granules compared to non-stimulated cells, both at culture days 7 (19 ± 3%, *n* = 5) and 21 (45 ± 4%, *n* = 5) (Fig. 2A and B). After a two-day resting period, mechanically stimulated (SS) Pm BM-MSCs still constitutively release more ATP to the extracellular fluid than the cells grown under resting conditions, when both groups were tested without any sort of stimulus for 30-min with PBS 1x. In fact, mechanically stimulated cell cultures (SS) grown for 21 days in an osteogenic-inducing medium release twice as much of ATP normalized by MTT values than that obtained in non-stimulated (CTR) cultures (Fig. 2C). These results also indicate that osteogenic-differentiated Pm BM-MSCs for 21 days release to the extracellular fluid higher ATP amounts than non-differentiated/proliferating cells cultured for 7 days (cf [29]).

### Mechanical stimulation (SS) does not account to slowdown the enzymatic breakdown of extracellular ATP and UDP by osteogenic differentiated Pm BM-MSCs

**Fig. 3:**
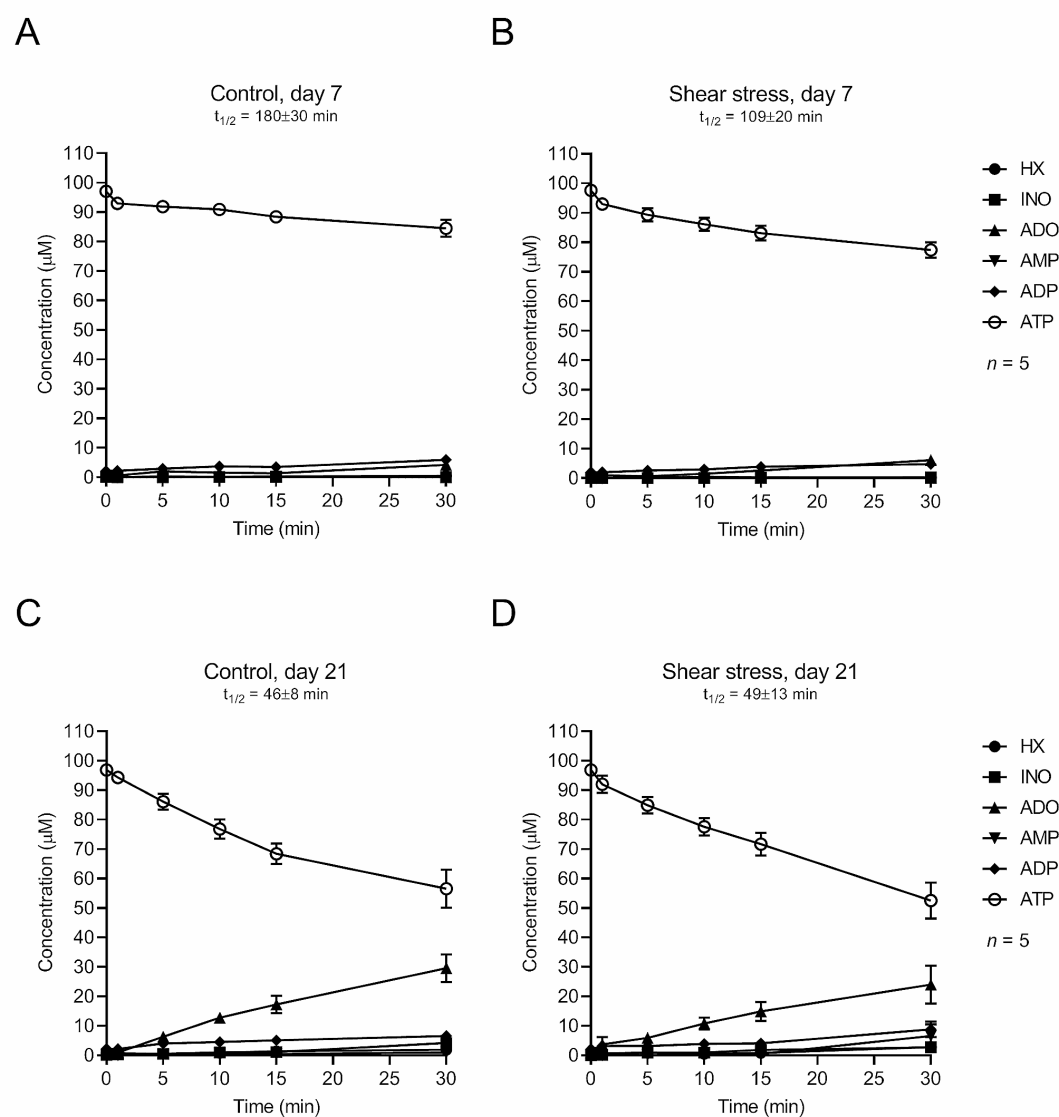
Mechanical stimulation (SS) does not significantly account to speed-up the extracellular breakdown of ATP by Pm BM-MSCs undergoing osteogenic differentiation. Shown is the time course of extracellular catabolism of ATP (100 µM) in BM-MSCs from Pm women grown for 7 (panels A and B) and 21 (panels C and D) days in an osteoblast-inducing medium obtained either in the absence or presence of shear-stress mechanical stimulation. ATP (100 µM) was added to the culture medium at time zero and samples were collected at the indicated times for HPLC analysis to quantify the substrate ATP and its metabolites: ADP, AMP, adenosine (ADO), inosine (INO) and hypoxanthine (HX). Each point represents mean ± S.E.M. of pooled data from 5 Pm women (71 ± 5 years old); cells from each individual were tested in duplicate. The calculated half-life time (t_1/2_) for each initial substrate is shown for comparison

Given our observation that ATP accumulates in the extracellular milieu of mechanically-stimulated (SS) Pm BM-MSC cultured for 21 days (Fig. 2C), we set to investigate whether this could be due to a slowdown of the enzymatic breakdown of extracellular nucleotides under such experimental conditions. Data show that the ability of non-stimulated Pm BM-MSCs (CTR) to hydrolyse extracellular ATP (100 µM) raises from culture day 7 (t½ 180 ± 30 min; Fig. 3A) to 21 (t½ 46 ± 8 min; Fig. 3C). Mechanical stimulation of Pm BM-MSCs (SS) transiently speedup (*P* < 0.05) the extracellular ATP hydrolysis at culture day 7 (t½ 109 ± 20 min; Fig. 3B), but this difference was abolished when comparing the extracellular breakdown of ATP (100 µM) between stimulated (SS) and non-stimulated (CTR) cells at culture day 21 (t½ 49 ± 13 min; Fig. 3D). ATP (100 µM) was sequentially hydrolysed into adenosine (ADO) both in SS and CTR cells, with almost no accumulation of inosine (INO) and hypoxanthine (HX), given the very low ecto-adenosine deaminase (ADA) activity normally present in these cells [18]. Very small amounts of AMP were also observed in the incubation fluid, since BM-MSCs undergoing osteogenic differentiation exhibit high ecto-5´-nucleotidase/CD73 activity, which is responsible for the fast dephosphorylation of AMP into ADO [18].

**Fig. 4:**
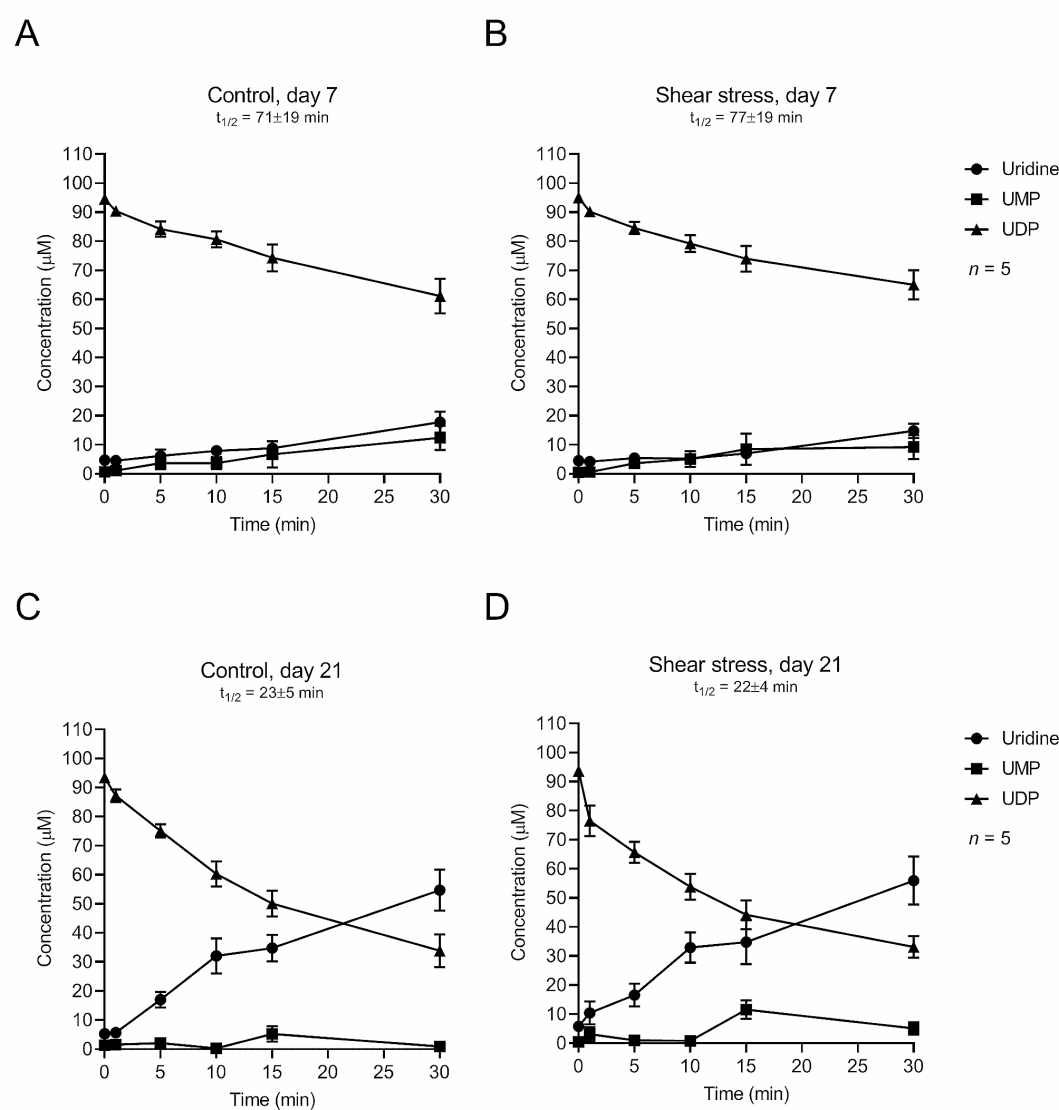
Mechanical stimulation (SS) does not significantly account to speed-up the extracellular breakdown of UDP by Pm BM-MSCs undergoing osteogenic differentiation. Shown is the time course of extracellular catabolism of UDP (100 µM) in human Pm BM-MSCs grown for 7 (panels A and B) and 21 (panels C and D) days in an osteoblast-inducing medium either in the absence or presence of shear-stress mechanical stimulation. UDP (100 µM) was added to the culture medium at time zero and samples were collected at the indicated times for HPLC analysis to quantify the substrate UDP and its metabolites: UMP and uridine. Each point represents mean ± S.E.M. of pooled data from 5 Pm women (71 ± 5 years old); cells from each individual were tested in duplicate. The calculated half-life time (t_1/2_) for each initial substrate is shown for comparison

Taking into consideration our interest in testing the osteogenic role of the P2Y_6_ receptor, we also set to investigate the influence of mechanical stimulation (SS) on the extracellular catabolism of its native ligand, UDP, by Pm BM-MSCs. Similarly to ATP (100 µM), the ability of non-stimulated Pm BM-MSCs in culture to hydrolyse extracellular UDP (100 µM) increases as cells differentiate from culture day 7 (UDP t½ 71 ± 19 min; Fig. 4A) to 21 (UDP t½ 23 ± 5 min; Fig. 4C). Mechanical stimulation (SS) did not significantly (*P* < 0.05) change the kinetics of the extracellular UDP catabolism by Pm BM-MSCs measured both in undifferentiated/proliferating (day 7; Fig. 4B) and osteogenic differentiated (day 21; Fig. 4D) stages. UDP metabolites included UMP and uridine, as previously reported under resting conditions by our group [18].

### Mechanical stimulation (SS) favours the osteogenic differentiation and formation of bone nodules by Pm BM-MSCs in culture via tonic activation of P2X7 and P2Y _6_ receptors

Previous findings from our group showed that the osteogenic commitment associated to tonic activation of P2X7 and P2Y_6_ receptors was significantly impaired in BM-MSCs originated from Pm women [8, 9], an effect that has been mainly attributed to overexpression of NTPDase3 [10]. Thus, under resting conditions, excessive breakdown of extracellular nucleotides by these cells abrogated the characteristic inhibitory effect of selective P2X7 and P2Y_6_ receptor blockage by A438079 (3 µM) and MRS 2578 (0.1 µM), respectively, in osteogenic differentiating BM-MSCs from Pm women.

**Fig. 5:**
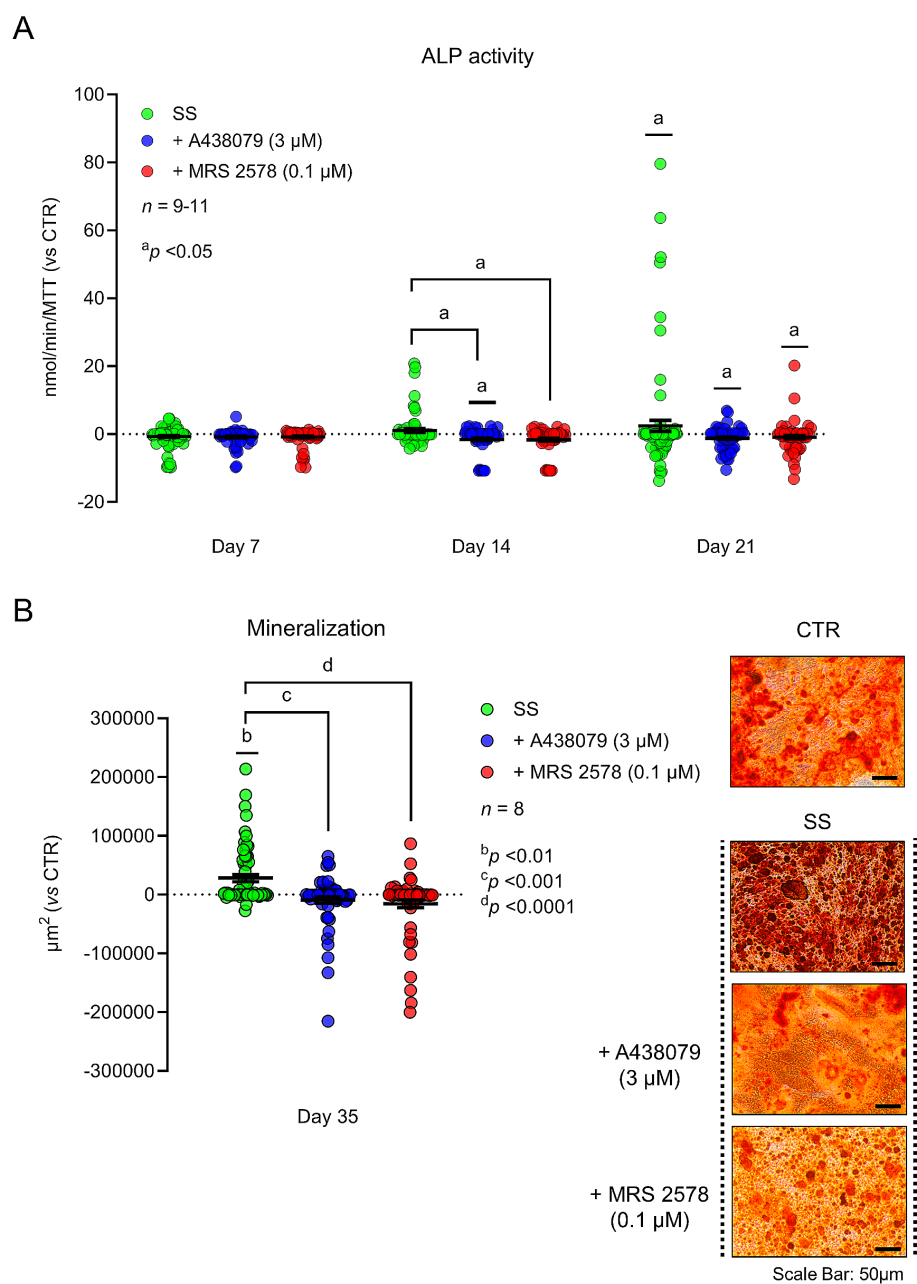
Mechanical stimulation facilitates the osteogenic differentiation and mineralization of cultured BM-MSCs from Pm women via the activation of P2X7 and P2Y_6_ receptors. BM-MSCs grown in an osteoblast-inducing medium for 7, 14, 21 and 35 days, either in the absence or presence of shear-stress mechanical stimulation. Mechanically-stimulated Pm BM-MSCs were also tested in the absence (SS group) and in the presence of selective P2X7 (SS + A438079, 3 µM) and P2Y_6_ (SS + MRS 2578, 0.1 µM) receptor antagonists. **Panel A** shows the ALP activity represented in nmol/min/MTT. Zero represents identity between represented conditions and the ALP activity obtained in non-stimulated (CTR) cells (horizontal dash line); control ALP activity values were 3.5 ± 0.4, 4.3 ± 1.0, 4.6 ± 0.6 nmol/min/MTT on days 7, 14 and 21, respectively. Scatter dot plot (with mean ± S.E.M.) represent pooled data from 11 Pm women (71 ± 3 years old); seven to sixteen replicates were performed per individual. ^a^*P*<0.05 (non-parametric Kruskal-Wallis test with uncorrected Dunn’s test) represent significant differences. **Panel B** shows the extracellular matrix mineralization at culture day 35. Zero represents identity between represented conditions and the total mineralized area values obtained in non-stimulated (CTR) cells (horizontal dash line); the total mineralized area in control cultures was 64,972 ± 14,792 µm^2^. Scatter dot plot (with mean ± S.E.M.) represent pooled data from 8 Pm women (70 ± 3 years old); four to eight replicates were performed per individual. ^b^*P*<0.01, ^c^*P*<0.001 and ^d^*P*<0.0001 (non-parametric Kruskal-Wallis test with Dunn’s multiple comparison test) represent significant differences. Right hand-side images show typical micrographs displaying bone nodule formation (red-brownish spots) in BM-MSC cultures from a Pm woman submitted (SS) or not (CTR) to mechanical stimulation either in the absence or presence of selective P2X7 (SS + A438079, 3 µM) and P2Y_6_ (SS + MRS 2578, 0.1 µM) receptor antagonists. Scale bar is 50 μm

**Fig. 6:**
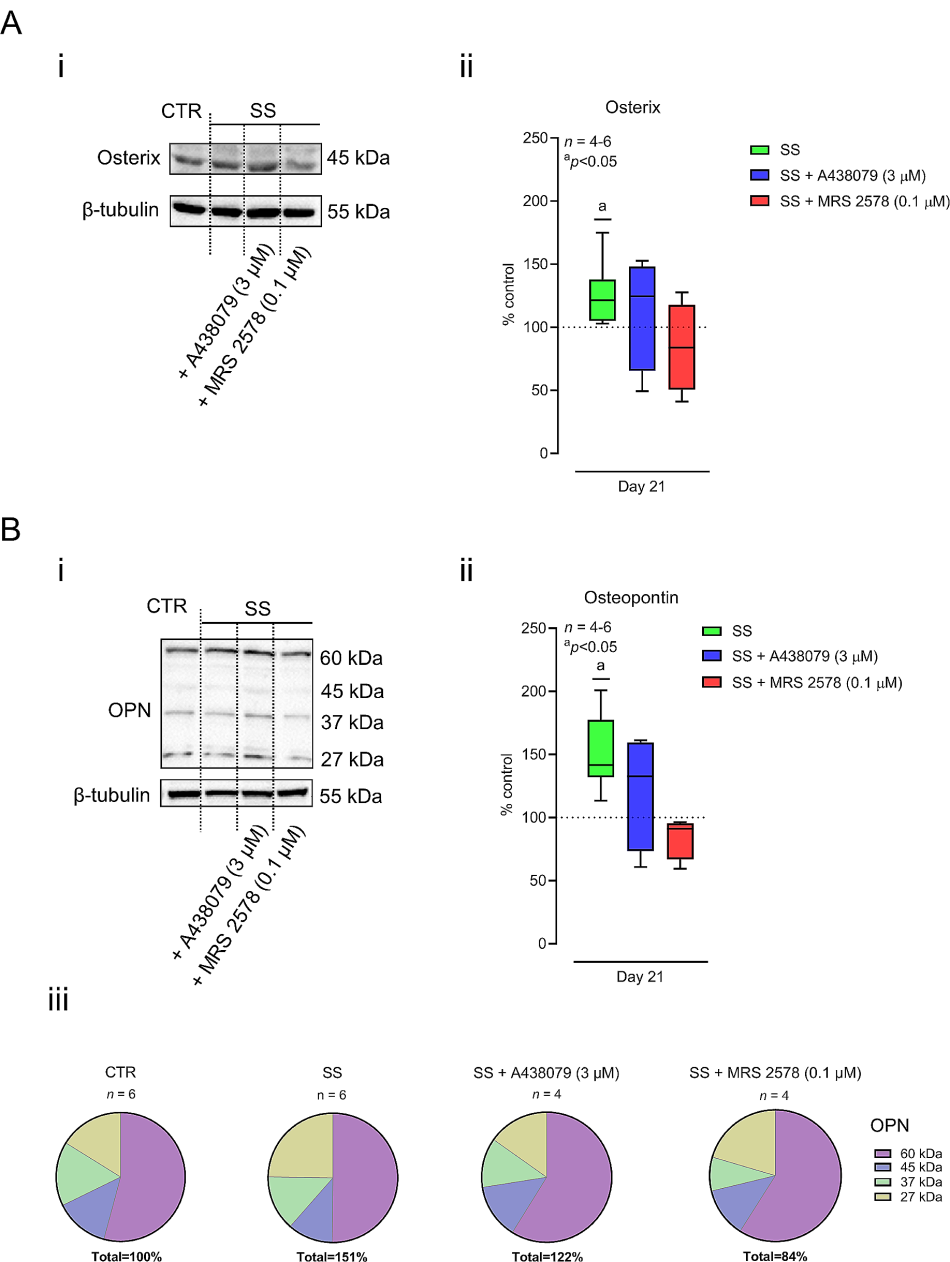
Mechanical stimulation (SS) increases the amount osteogenic transcription factors, osterix and osteopontin (OPN), in Pm BM-MSCs via a mechanism dependent on tonic P2X7 and P2Y_6_ receptors activation. BM-MSCs grown in an osteoblast-inducing medium for 21 days, either in the absence or presence of shear-stress mechanical stimulation. Mechanically-stimulated Pm BM-MSCs were also tested in the absence (SS group) and in the presence of selective P2X7 (SS + A438079, 3 µM) and P2Y_6_ (SS + MRS 2578, 0.1 µM) receptor antagonists. **Panels Ai and Bi**, show typical immunoblots stained for osterix (45 kDa) and OPN (60 − 27 kDa) transcription factors of cultured BM-MSCs obtained from Pm women with 70 and 57 years-old, respectively; β-Tubulin (55 kDa) was used as a house-keeping gene product standard for normalization purposes. Uncropped full-length gel blots may be found in Supplementary Fig. 2. **Panels Aii and Bii** show the relative amounts of indicated transcription factors at culture day 21. The results are in percentage of non-stimulated cells (CTR) of the same individual, considered as 100% for normalization purposes (dashed horizontal line). The relative protein amounts in non-stimulated cells were 0.27 ± 0.04 for osterix/β-Tubulin and 1.8 ± 0.4 for OPN/β-Tubulin. Boxes and whiskers represent pooled data from 4 to 6 Pm women (66 ± 4 years old); ^a^*P*<0.05 (non-parametric Wilcoxon matched-pairs signed rank test, considering 100% for the CTR situation) represents significant differences. **Panel Biii** shows the relative protein amount of each OPN cleavage products (60, 45, 37 and 27 kDa) obtained in all tested subgroups

Here, we show that mechanical stimulation (SS) increases the density of P2X7 and P2Y_6_ receptors and the outflow of ATP from cultured Pm BM-MSCs undergoing osteogenic differentiation. This prompted us to test whether mechanical stimulation (SS) could rehabilitate the P2X7 and/or P2Y_6_ receptors-operated osteogenic commitment of cultured Pm BM-MSCs. Mechanical stimulation (SS) promoted the osteogenic differentiation of cultured Pm BM-MSCs; this was measured as increases (i) in ALP activity normalized by the cells proliferation/viability (MTT values) (Fig. 5A) and (ii) in the amount of osterix and osteopontin transcription factors as cells differentiate (Fig. 6). In our hands, mechanical stimulation (SS) of the cultures did not increase the proliferation/viability of the Pm BM-MSCs, since no differences were observed in the MTT values among stimulated and non-stimulated cell groups (Supplementary Fig. 1). Yet, stimulated (SS) cultures exhibited increased ability to form mineralized bone nodules (Alizarin Red staining) when the cultures were prolonged until day 35 (Fig. 5B).

Selective blockage of P2X7 and P2Y_6_ purinoceptors with A438079 (3 µM) and MRS 2578 (0.1 µM), respectively, prevented the osteogenic commitment (Figs. 5A and 6) and the formation of bone nodules (Fig. 5B) of cultured Pm BM-MSCs. These findings suggest that mechanical stimulation (SS) rehabilitates the osteogenic differentiation of BM-MSCs from Pm women via tonic activation of P2X7 and/or P2Y_6_ receptors.

### Xenotransplantation of mechanically stimulated (SS) Pm BM-MSCs accelerates osteointegration and bone repair in an “in vivo” animal model

**Fig. 7:**
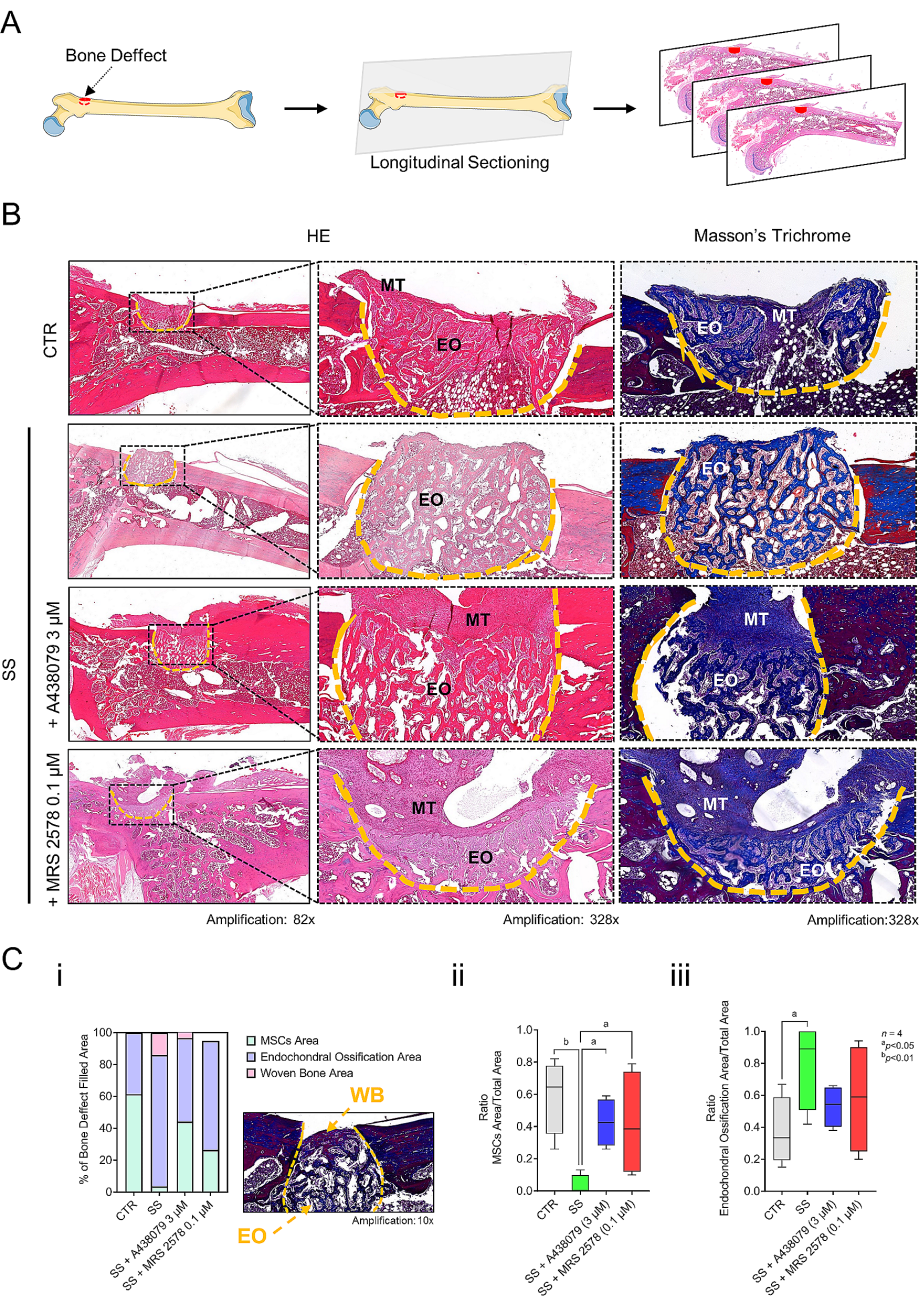
Transplanted BM-MSCs submitted to mechanical stimulation (SS) accelerate closure of critical bone defects in an “in vivo” animal model compared to non-stimulated cells and this effect depends on tonic P2X7 and P2Y_6_ receptors activation. **Panel A** shows a schematic representation of a rat femur with a critical bone defect drilled in the greater trochanter prepared to receive the encapsulated Pm BM-MSCs (for details see Materials and Methods). **Panel B** shows representative micrograph sections of critical bone defects in rat femora filled with collagen I-encapsulated BM-MSCs of a 60-years old woman. Before xenotransplantation, BM-MSCs were differentiated using an osteoblast-inducing medium for 21 days in the absence (CTR) or presence of shear stress (SS) mechanical stimulation only or together with of selective P2X7 (SS + A438079, 3 µM) and P2Y_6_ (SS + MRS 2578, 0.1 µM) receptor antagonists. Shown are images stained with hematoxylin and eosin (HE) and Masson’s trichrome highlight the bone defect area (dashed yellow line). The mesenchymal tissue (MT) consists of BM-MSCs transplanted and/or recruited to the injury site; endochondral ossification (EO) is evidenced as the result of secondary bone repair. **Panel C** shows three graphs computed from histological digital images, representing: (i) the percentage of MSCs area, endochondral ossification area and woven bone area as a function of the bone defect filled area (100%); (ii) the ratio between the area occupied by BM-MSCs and the total bone defect area; and (iii) the ratio between the endochondral ossification area and the total bone defect area, obtained for each experimental condition. Boxes and whiskers represent pooled data from 4 individual experiments; the cells used were obtained from 4 Pm women (60, 68, 76 and 79 years old); *P < 0.05 (ordinary one-way ANOVA with Fisher’s LSD test) represents significant differences. The insert part in panel Ci shows a higher magnification (410x) of a micrograph section stained with Masson’s trichrome in which the bone defect area shows spicules of newly formed woven bone (WB) through endochondral ossification (EO)

Xenotransplantation of collagen I-encapsulated BM-MSCs from Pm women, which have not been submitted to mechanical stimulation (CTR), partially filled the bone defect area with the following proportion: 61 ± 12% MSCs and 39 ± 12% endochondral ossification (EO) (Fig. 7B and Ci). The area of EO increases to 82 ± 13% when the femoral bone defects were xenotransplanted with mechanically stimulated (SS) Pm BM-MSCs and, consequently, the critical bone defects show spicules of newly formed woven bone (WB) occupying a previously unrecognized area of about 14 ± 14% (Fig. 7B and C, high magnification inset). All these changes were fully reversed to CTR levels when the mechanically stimulated (SS) cells were concurrently treated with P2X7 or P2Y_6_ receptor antagonists, respectively A438079 (3 µM) and MRS 2578 (0.1 µM). In the presence of the P2 purinoceptor antagonists, the proportion of MSCs were 44 ± 8% and 42 ± 17% and the total EO area were 54 ± 7% and 58 ± 17%, respectively, thus indicating a delay in WB formation (Fig. 7B and C).

As expected, providing that the osteogenic commitment of BM-MSCs from Pm women is severely compromised [8, 9], no significant changes were detected in the repair of critical bone defects among sham-operated femora (no transplanted cells), collagen I-loaded (Col I) and xenotransplanted defects with non-stimulated Pm BM-MSCs (CTR) (Supplementary Fig. 3). Overall, these findings suggest that mechanically stimulated (SS) BM-MSCs from Pm women accelerate osteointegration and the histological repair of critical bone defects in the living rat through a mechanism that depends on the tonic activation of P2X7 and/or P2Y_6_ purinoceptors sensitive to ATP and UDP, respectively.

## Discussion

Understanding the impact of biomechanical challenges in the expansion and osteogenic differentiation of BM-MSCs is critical in bone tissue engineering to emulate the most adequate “in vitro” cellular environment, ultimately ensuring higher success rates for “in vivo” bone repair [30]. Because we aimed at investigating, whether “in vitro” mechanical stimulation (SS) of BM-MSCs from Pm women could rehabilitate their osteogenic commitment and if xenotransplantation of these cells could contribute to bone repair in an “in vivo” animal model, we used in this study a previously validated experimental protocol [22, 23]. The works of Delaine-Smith and Reilly [22] and Zhou et al. [23] predict that a seesaw system can produce low-magnitude fluid-shear stress (SS) in standard culture dishes/plates and that this promotes osteogenesis in “in vitro” BM-MSCs cultures.

Data show here for the first time that “in vitro” mechanical stimulation (SS) of BM-MSCs can partially rehabilitate the lost osteogenic commitment of such cells in Pm women, which may ultimately readmit their osteointegration and bone repair potential via mechanisms depending on tonic activation of P2X7 and/or P2Y_6_ purinoceptors. Using a non-drug approach method, we demonstrated that mechanically stimulated (SS) Pm BM-MSCs (i) have increased ability to accumulate and release ATP, and (ii) overexpress ATP-sensitive P2X7 and UDP-sensitive P2Y_6_ purinoceptors during the proliferative/expansion and osteogenic differentiated culture stages, respectively. These features definitively contribute to foster the osteogenic commitment of mechanically stimulated (SS) Pm BM-MSCs, measured as increases (i) in the ALP activity, without any measurable effects on cells proliferation/viability, (ii) in the amount of osteogenic transcription factors (osterix and osteopontin), and (iii) in the ability to form mineralized bone nodules, compared to non-stimulated cells of the same individuals. Using an “in vivo” animal model, we proved that mechanical stimulation (SS) of BM-MSCs from Pm women gained the ability to accelerate the histological repair of critical bone defects after xenotransplantation and convenient osteointegration. Remarkably, all the osteogenic inducing effects promoted by mechanical stimulation (SS) of Pm BM-MSCs were dismissed by blocking P2X7 or P2Y_6_ purinoceptors with A438079 (3 µM) and MRS 2578 (0.1 µM), respectively, thus confirming the paramount role of these two purinoceptor osteogenic promotors [8–10]. Although Pm BM-MSCs exhibit immunoreactivity against metabotropic P2Y_2_, P2Y_4_, and P2Y_6_ receptors, the selective activation of UDP-sensitive P2Y_6_ receptors explains the observed intracellular Ca^2+^ oscillations and the osteogenic differentiating properties of uracil nucleotides in Pm BM-MSCs in culture. The lack of effect of the stable UTP analogue, UTPγS, in Pm BM-MSCs undergoing osteogenic differentiation practically excludes the involvement of P2Y_2_ and P2Y_4_ receptor-mediated effects in these cells [8].

Purinergic signalling results from complex interactions between nucleotides release sites, ecto-nucleotidases, purinoceptors and second messenger pathways, which co-existence and relative activity fine-tuning regulate signal adaptations to cell functioning. Concerning the osteogenic commitment of MSCs derived from the human BM, our group showed that high enough extracellular levels of ATP and UDP are critical to promote the osteogenic activity of P2X7 and P2Y_6_ purinoceptors in these cells [8, 9]. Yet, this threshold is not reached in cells originated from Pm women, mostly because these Pm BM-MSCs overexpress NTPDases leading to the excessive breakdown of released adenine and uracil nucleotides and, thus, failure of this cells to differentiate into bone-forming osteoblasts [8–11]. The presence of NTPDase2 in the plasma membrane of human BM-MSCs explains the rapid conversion of released UTP into UDP and, subsequently, the putative activation of the P2Y_6_ receptor subtype in these cells [8, 31]. Dephosphorylation of nucleotide triphosphates into their diphosphate-derivatives is faster in more differentiated (less proliferative) human BM-MSCs undergoing osteogenic differentiation, a situation also complying with the increased expression of NTPDases1, 2, and 3 in differentiated later stages of Pm BM-MSC cultures. Among all these ecto-enzymes, the NTPDase3 emerges as a major determinant, given that it is highly overexpressed in BM-MSCs from Pm women compared to the cells of younger females and age-matched men [8, 10]. Despite the exact trigger of NTPDase3 overexpression in Pm BM-MSCs is still elusive one may suspect of oestrogen-deficiency, but there are no clinical trials either proving or rejecting this hypothesis given the difficulty to obtained controlled samples. Nevertheless, we recently provided experimental evidence showing that selective blockage of NTPDase3 with antraquinone derivative, PSB 06126, or the monoclonal antibody, hN3-B3s, and the NTPDase3 gene silencing with a selective shRNA, led to extracellular ATP accumulation and to osteogenic differentiation and mineralization of Pm BM-MSC cultures via the activation of P2X7 and P2Y_6_ receptors [10].

Regardless of these novel insights implicating the manipulation of NTPDase3 expression and activity as a putative therapeutic target to treat bone mass loss in aged women, here we found only mild (non-significant) differences in the extracellular breakdown of ATP and UDP among mechanically stimulated (SS) osteogenic-primed Pm BM-MSCs and non-stimulated cells of the same individuals (see Figs. 3 and 4). Data suggest that, in our hands, mechanical stimulation (SS) is not enough to reverse NTPDases overexpression and excessive breakdown of extracellular ATP and UDP in Pm BM-MSCs undergoing osteogenic differentiation. Thus, the beneficial effects of mechanical stimulation (SS) on the osteogenic commitment of Pm BM-MSCs must come from increases in the intracellular accumulation and release of adenine (and uracil) nucleotides and/or from the boost in P2X7 and P2Y_6_ purinoceptors density in the plasma membrane.

Electromagnetic field therapy has been used to treat bone fractures and musculoskeletal disorders, including osteoarthritis and rheumatoid arthritis, by directly inducing or accelerating the osteogenic differentiation of BM-MSCs associated to overexpression of P2X7 receptors and PKB/GSK3β/β-catenin downstream signalling activation [32]. After close characterization of the *P2RX7* gene promoter, Bergamin et al. [33] identified several specific binding sites for transcription factors differently involved in the mineralization process. In particular, they have demonstrated that the P2X7 receptor is upregulated by the nuclear factor of activated T cells cytoplasmic 1 (NFATc1), a member of the NFAT family of cytosolic Ca^2+^ dependent transcription factors that is known to be upregulated by mechanical stimulation in bone cells. Moreover, Riddle et al. [34] proved that ATP is necessary for fluid flow–induced proliferation of BM-MSCs from an 18-year-old male donor, acting by triggering the mobilization of intracellular calcium, activating calcineurin, and stimulating the nuclear translocation of NFATc1. Unexpectedly, we found no changes in the proliferation/viability (MTT assay) performed in mechanically stimulated BM-MSCs originated from Pm women compared to non-stimulated cells of the same individuals (Supplementary Fig. 1), most likely due to age and gender of the donors, as well as to putative differences in experimental protocols. In our hands, P2X7 receptors activation fosters the osteogenic differentiation and mineralization of Pm BM-MSCs cultures through mechanisms involving intracellular Ca^2+^ oscillations and reversible plasma membrane cytoskeleton rearrangements (zeiosis) associated to PLC/PKC/Rho-kinase activation [9].

Mechanically stimulated (SS) Pm BM-MSCs undergoing osteogenic differentiation accumulate higher amounts of ATP in fluorescently labelled quinacrine-stained granules than non-stimulated cell cultures of the same individuals (Fig. 2A and B). Biomechanical forces, like stretching, compression or shear stress, influence many cellular processes including cell division, migration, gene expression, morphogenesis, and cell adhesion. Mitochondrial biogenesis and metabolic shifts are also crucial mechanisms accounting to stem cells differentiation [35]. Differentiated cells display a more developed and functional mitochondrial network that rely on oxidative phosphorylation and increased ATP synthesis [36]. Mechanical stimulation (SS) significantly increases the expression of key genes related to mitochondrial biogenesis and quality control, as well as mtDNA content and mitochondrial mass [37]. In this context, one may speculate about increases in mitochondrial biogenesis and activity to explain the intracellular ATP accumulation in mechanically stimulated BM-MSC cultures, considering that this normally participates in cells proliferation and osteogenic differentiation [38]. This hypothesis is strengthened given that differentiating cells display a shift from the anaerobic glycolytic metabolism towards the more effective mitochondrial oxidative metabolism [39]. Mitochondria are maintained at a relatively low activity level in resting BM-MSCs, while upon activation the number of mtDNA copies, the levels of the protein involved in the respiratory chain, the oxygen consumption rate, the levels of mRNA transcripts encoding to mitochondrial biogenesis, and the intracellular ATP content, are all increased [35]. Likewise, there is a negative interaction between oxidative stress and skeletal integrity [40, 41].

Compression forces release ATP from BM-MSC osteoprogenitors and osteoblasts to promote osteogenesis in bone niches [42]. Romanello et al. [43] first noticed controlled ATP release from human osteoblasts; since then, this has been a widely investigated subject. Here, we show that mechanically stimulated (SS) Pm BM-MSCs intrinsically release higher amounts of ATP to the extracellular milieu than non-stimulated cells; this phenomenon has been noticed without any sort of stimulation during measurements and was still detectable 2 days after the last mechanical challenge (Fig. 2C). We also proved that mechanical stimulation (SS) made only twice a week was sufficient to prime Pm BM-MSCs to accumulate intracellular ATP and to promote the constitutive release of nucleotide to the extracellular milieu. These mild stimulation conditions are noteworthy, as they dramatically differ from the harsh mechanical stimulation conditions (e.g. longer stimulation periods, no resting washout periods, constitutive vs. evoked ATP release) found in previous reports [44, 45].

Data in the literature point towards vesicle exocytosis as the primary mechanism responsible for the evoked release of ATP from osteoblasts [29, 43, 46]. Maxi-anion channels [47], hemichannels containing connexin-43 and pannexin-1 [48], and the ionotropic P2X7 receptor pore [49], may also contribute to the release of ATP from bone cells. Brandao-Burch and colleagues [49] demonstrated that selective inhibitors of vesicular exocytosis, including *N*-ethylmaleimide (NEM) and brefeldin A, had no effect on ATP release from osteoblasts, whereas several selective P2X7 receptor antagonists inhibited ATP release via different pharmacological mechanisms without affecting the viability of the cells. As aforementioned, ATP release into the bone microenvironment may also occur via hemichannels containing connexin-43 and pannexin-1 [43, 47, 50]. Unpublished observations from our group showed no changes in the connexin-43 immunoreactivity between mechanically stimulated (SS) and non-stimulated Pm BM-MSCs throughout the entire culture period (data not shown). Clearly, the mechanism(s) involved in ATP release from BM-MSCs undergoing osteogenic differentiation require thorough consideration of age and gender of the cells, as well as the stimulation conditions used to perform the experiments, but these requirements are far beyond the scope of this study.

This study provides compelling evidence that mechanical stimulation (SS) prime Pm BM-MSCs to rehabilitate their osteogenic potential and capacity of osteointegration to increase bone repair compared to non-stimulated cells of the same individual. This is achieved by favouring adenine (and uracil) nucleotides release and, consequently, boosting tonic activation of P2X7 and P2Y_6_ purinoceptors. The osteogenic commitment of mechanically primed Pm BM-MSCs was demonstrated by progressive increases in multiple osteogenic differentiation biomarkers at culture day 21, namely the ALP activity, the amount of the protein content of the osteogenic transcription factors (osterix and osteopontin), and the ability to form mineralized bone nodules, compared to non-stimulated cells of the same individuals. All these changes were abrogated by concurrently treating mechanically stimulated (SS) cell cultures with selective P2X7 and P2Y_6_ purinoceptors, respectively A438079 and MRS 2578, but these antagonists were devoid of effect in non-stimulated cell cultures simply because Pm-originated BM-MSCs lack their characteristic osteogenic potential under the present experimental conditions (see [8, 9]). While differences were significant among P2X7 and P2Y_6_ receptors immunoreactivity between proliferating (P2X7 > P2Y_6_, at culture day 7) and fully differentiated (P2X7 < P2Y_6_, at culture day 21) cells, this had no substantial repercussion in the blocking effects of the two receptor antagonists, A438079 and MRS 2578, providing that the drugs were present during the entire culture period. The most obvious explanation for these findings is that both ionotropic P2X7 and metabotropic P2Y_6_ receptors synergize at different culture stages (e.g. day 7 vs. day 21) to foster the osteogenic differentiation of mechanically stimulated (SS) Pm BM-MSCs, most probably by using different intracellular Ca^2+^ signatures and downstream second messenger pathways [8, 9].

Bone formation depends on the expression of transcription factors, like osterix [51, 52]. Therefore, mechanical stimulation of bone osteoprogenitors, such as BM-MSCS, might have a profound influence on bone remodelling, while disuse or lack of mechanical loading accelerates bone resorption [53], like that occurring in astronauts spending extended periods of time (at least 6 months) in weightless environments. There is a gap in our knowledge concerning the purinergic signalling mechanisms in such harsh conditions. Yet, irisin, an exercise- and/or cold-induced myokine, contributes to maintain the osteoblast phenotype and preserves these cells from the significant weightlessness-induced decline of their characteristics via increases in the expression of Runx-2 and osterix transcription factors [54]. Likewise, extracellular nucleotides released in response to mechanical stress fosters the activation of Runx-2 DNA-binding activity along with increased osterix expression, via a mechanism involving PKC and distinct mitogen-activated protein kinase cascades [55, 56]. The interplay between irisin and the purinergic signalling transcriptomics is not new, but deserves to be further elucidated in bone diseases, as occurring in obesity and related metabolic disorders [57]. Like that observed regarding overexpression of osterix protein amounts following the mechanical stimulation of Pm BM-MSCs, the levels of Runx-2 also increase under similar experimental conditions (unpublished preliminary results).

Concerning osteopontin (OPN), a sialoprotein with high calcium binding capacity deeply involved in mineralization, it accounts for about 2% of the non-collagen proteins in the bone marrow [58, 59]. OPN is mainly secreted by osteoblasts, but it can also be produced by osteoprogenitor-like BM-MSCs, as well as by other hematopoietic cells, in the bone marrow. The abnormal expression of OPN is involved in the development of skeleton diseases such as osteoporosis, rheumatoid arthritis, and osteosarcoma [60]. mRNA transcripts and protein levels of collagen 1, Runx-2, OPN and ALP increased in periodontal ligament stem cells during osteogenic differentiation triggered by repetitive mechanical stretch [61]. Likewise, dynamic compression upregulates mRNA transcription and the protein levels of Runx-2, BMP-2 and OPN in osteogenic differentiating MC3T3-E1 cells [62]. Our study agrees with previous findings showing that OPN protein amounts increase in cells submitted to mechanical stimulation. OPN is a negatively charged heavily phosphorylated extracellular matrix protein. It is composed of about 300 amino acids and is expressed as a 33-kDa native protein, which exhibit many cleavage and post-translational modification sites, thus changing its apparent molecular weight in the immunoblots ranging from about 27 to 60 kDa (see Fig. 6B). Runx-2 and osterix transcription factors are required for the expression of OPN, yet the functional role of OPN proteolytic fragments is almost completely unexplored [63]. Here, we found no significant differences in the relative amounts of OPN-related protein species among CTR and SS BM-MSCs (Fig. 6Biii).

Bone remodels either by direct intramembranous (primary) or by indirect (secondary) fracture healing. In large bone defects, such as following surgical ostheosynthesis of weight bearing limbs subject to micro motion stimulation, the indirect fracture healing consists of both intramembranous and endochondral bone formation [64]. There are four overlapping stages occurring in secondary bone repair. These stages comprise: (1) the inflammatory response, formation of the hematoma and BM-MSCs recruitment; (2) the soft callus formation, through endochondral and intramembranous ossification and angiogenesis; (3) the hard callus formation, defined by osteogenesis and woven bone formation taking place after chondrocytes apoptosis; and (4) the resorption of the woven bone and the formation of cortical and trabecular bone [28]. Our findings show that xenotransplantation of mechanically-primed collagen I-encapsulated Pm BM-MSCs into critical bone defects increase within 10 days the relative area occupied by endochondral bone formation and woven bone formation, while decreasing the early-stage area of resident BM-MSCs, compared to the situation where non-stimulated cells and/or only collagen I scaffolds were used. Mechanically-stimulated cells also favoured the appearance of scattered hypertrophied chondrocytes trapped within the calcified matrix corresponding to later stages of endochondral ossification, as well as the observation of small areas of lamellar bone dispersed between the woven bone indicating the beginning of the remodelling process. The endochondral ossification and woven bone formation was significantly delayed after xenotransplantation of mechanically stimulated Pm BM-MSCs treated with A438079 and MRS 2578 to prevent the tonic activation of P2X7 and P2Y_6_ purinoceptors, respectively.

## Conclusion

Overall data suggest that in vitro mechanical stimulation rehabilitates the purinergic cell-to-cell communication fostering the osteogenic differentiation and osteointegration of BM-MSCs from Pm women. Insights from this study may prove useful for the development of new drug-free cell-based therapeutic approaches combining cell expansion and osteogenic rehabilitation of Pm BM-MSCs for diseases associated with bone mass loss, namely fractures due to osteoporosis and rheumatoid arthritis, and bone mal-union (pseudarthrosis).

### Electronic supplementary material

Below is the link to the electronic supplementary material.

**Supplementary Material 1: Suppl Fig. 1.** Growth/viability of BM-MSCs from 11 Pm women (71 ± 3 years old) grown in an osteoblastic inducing medium for 21 days. Boxes and whiskers represent cell viability/proliferation measured by the MTT assay; eight to sixteen replicates were performed per individual. Two-way ANOVA with Tukey’s test for multiple comparisons reveal no significant differences between control and mechanically-stimulated cells.

**Supplementary Material 2: Suppl Fig. 2.** High resolution images of original full-length uncropped immunoblots for Osterix (OSX, 33–45 kDa) and Osteopontin (OPN, 60–27 kDa) transcription factors using BM-MSC from 6 Pm women (53, 57, 67, 69, 70 and 80 years-old), which were cultured in an osteogenic-inducing medium in the absence or presence of mechanical stimulation. The cells from 4 out of 6 Pm women were also incubated with A438079 (3 µM, a selective P2X7 receptor antagonist) and MRS 2578 (0.1 µM, a selective P2Y_6_ receptor antagonist). The house keeping gene protein product, β-Tubulin (55 kDa), was used for normalization purpose. Dashed boxes indicate cropped regions of the blots depicted in Fig. 6 (panels A and B).

**Supplementary Material 3: Suppl Fig. 3.** Sham (no-cells) and collagen I (Col I)-loaded femoral defects used as controls for the in vivo experiments. **Panel A** shows representative micrograph sections of femoral defects stained with hematoxylin and eosin (HE) and Masson’s trichrome at different magnifications (82x and 328x) taken from sham (no-cells) and collagen I (Col I)-loaded defects used as controls of data shown in Fig. 7. Bone defects are outlined with a dashed yellow line. MT represents the mesenchymal tissue comprising autologous MSCs recruited to the injury site; EO denotes endochondral ossification as result of secondary bone repair. **Panel B** shows three graphs computed from histological digital images representing (i) the percentage of MSCs area, endochondral ossification area and woven bone area as a function of the bone defect-filled area (100%); (ii) the ratio between MSCs and bone defect total area; and (iii) the ratio between endochondral ossification and bone defect total area, for each experimental condition. Data obtained with transplanted BM-MSCs differentiated for 21 days in an osteoblast-inducing medium (CTR), is also shown for comparison. Boxes and whiskers represent pooled data from 4 individual experiments; the cells were obtained from the same 4 Pm women (60, 68, 76 and 79 years old) used in Fig. 7. Not significant (*ns*; ordinary one-way ANOVA with Fisher’s LSD test).

## Data Availability

The data that support the findings of this study are available from the corresponding author upon reasonable request.

## Abbreviations

A.u.: Arbitrary units
A438079: 3-[[5-(2,3-dichlorophenyl)-1 H-tetrazol-1-yl]methyl]pyridine hydrochloride
ADO: Adenosine
Alizarin red S: 3,4-dihydroxy-9,10-dioxo-2-anthracenesulfonic acid sodium salt
ALP: Alkaline phosphatase
BM-MSCs: Bone marrow-derived mesenchymal stem cells
Col I: Collagen I
EO: Endochondral ossification
HE: Hematoxylin-eosin
HPLC: High performance liquid chromatography
Hx: Hypoxanthine
INO: Inosine
LDH: Lactate dehydrogenase
Lenti-shRNA: Lentiviral short hairpin RNA
MRS 2578: N, N’’-1,4-butanediylbis[N’-(3-isothiocyanatophenyl) thiourea
MS: Mechanical stimulation
MTT: 3-[4,5-dimethylthiazol-2-yl]-2,5-diphenyltetrasodium bromide
OPN: Osteopontin
OSX: Osterix
PBS: Phosphate buffered saline
Pm: Post-menopausal
PNP: P-nitrophenyl phosphate
ROI: Regions of interest
SDS-PAGE: Sodium dodecyl sulphate-polyacrylamide gel electrophoresis
shRNA: short hairpin RNA
WB: Woven bone
α-MEM: α-Minimal essential medium
