# Supplementary figures and images for: Mechanical stimulation-induced purinome priming fosters osteogenic differentiation and osteointegration of mesenchymal stem cells from the bone marrow of post-menopausal women
Source: Stem Cell Res Ther. 2024 Jun 18;15:168. doi: 10.1186/s13287-024-03775-4 (PMC11184869; doi:10.1186/s13287-024-03775-4)

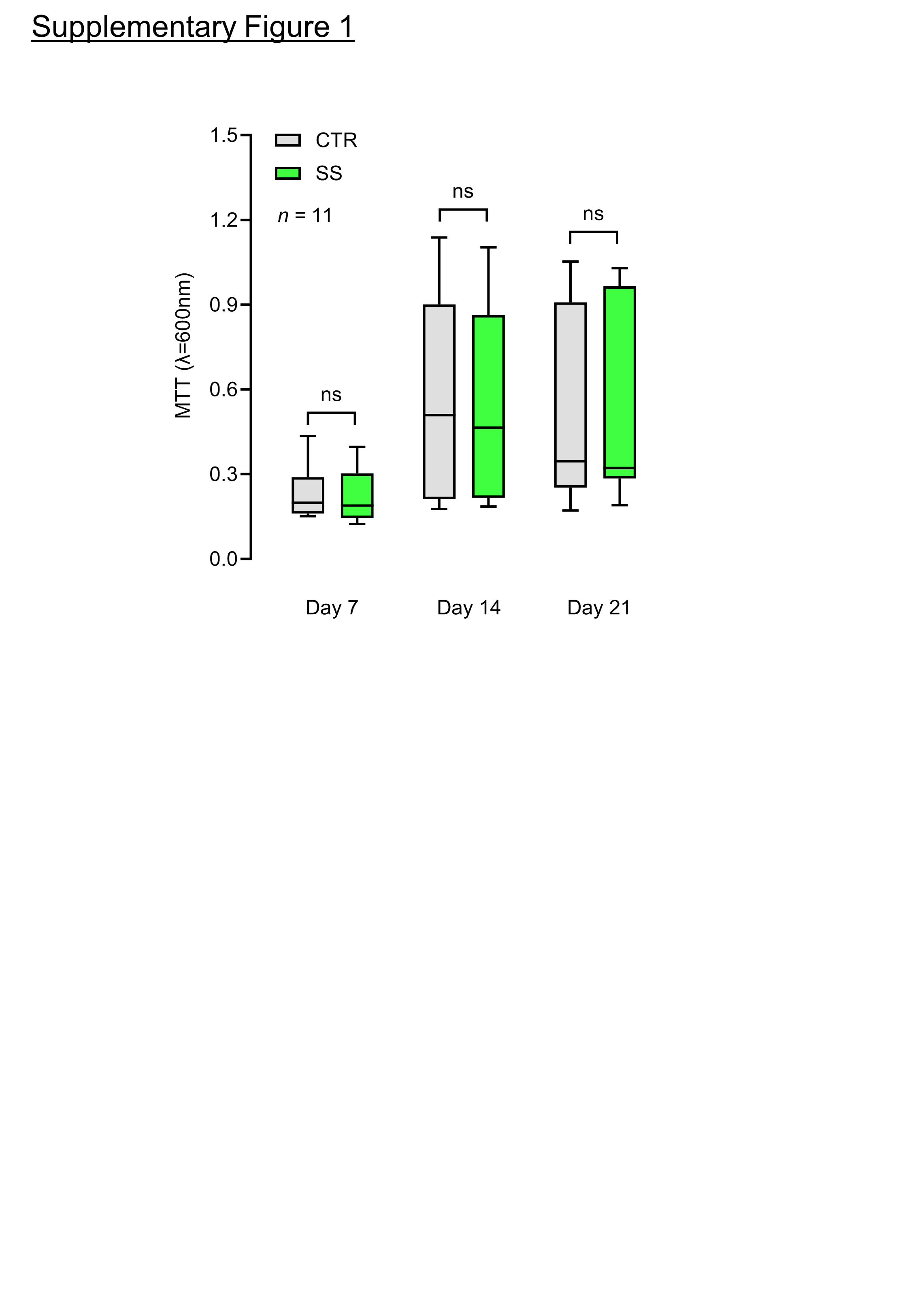

Supplement: Supplementary file 1 — Supplementary Material 1: Suppl Fig. 1. Growth/viability of BM-MSCs from 11 Pm women (71 ± 3 years old) grown in an osteoblastic inducing medium for 21 days. Boxes and whiskers represent cell viability/proliferation measured by the MTT assay; eight to sixteen replicates were performed per individual. Two-way ANOVA with Tukey’s test for multiple comparisons reveal no significant differences between control and mechanically-stimulated cells. [file 13287_2024_3775_MOESM1_ESM.tif]

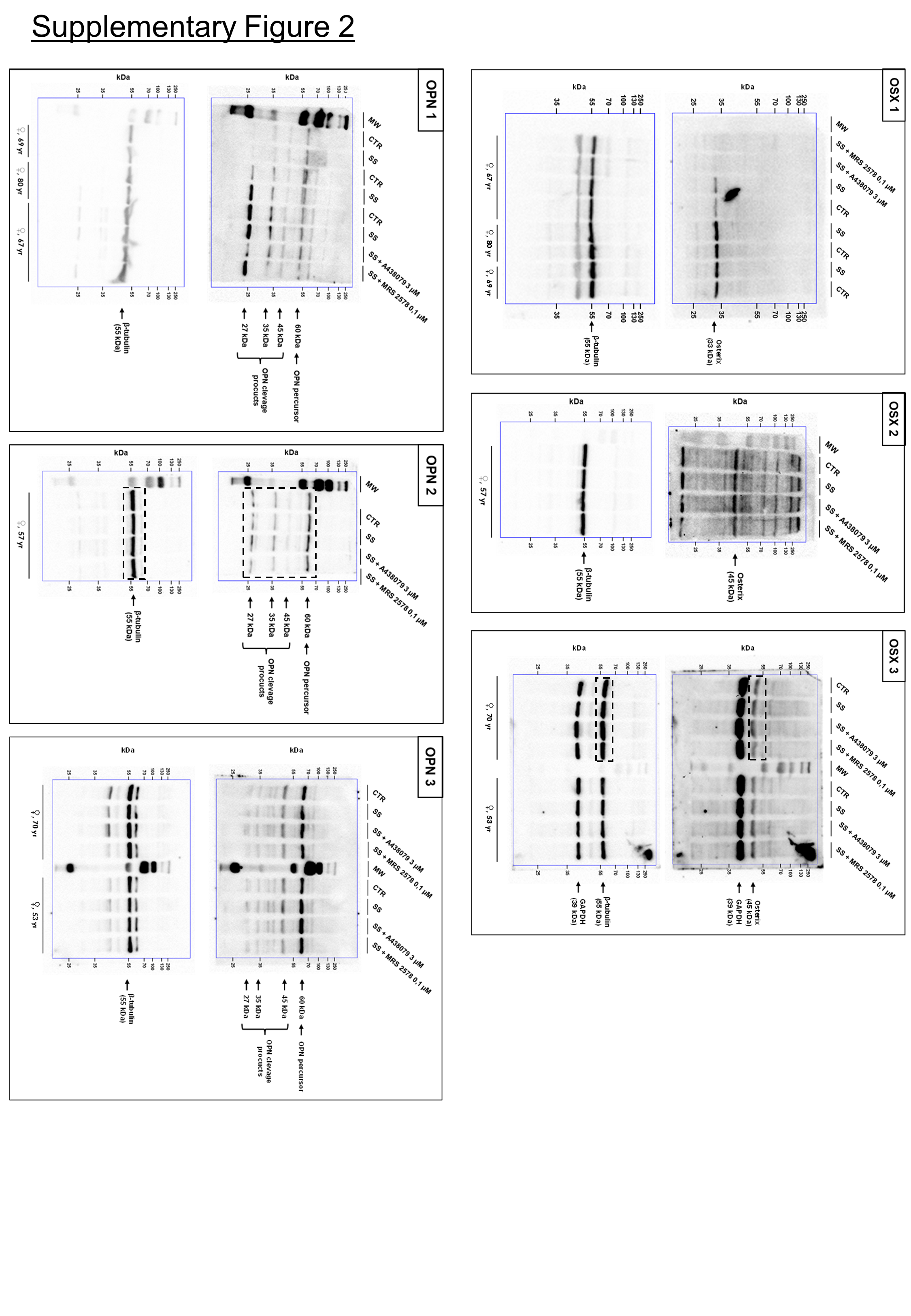

Supplement: Supplementary file 2 — Supplementary Material 2: Suppl Fig. 2. High resolution images of original full-length uncropped immunoblots for Osterix (OSX, 33–45 kDa) and Osteopontin (OPN, 60–27 kDa) transcription factors using BM-MSC from 6 Pm women (53, 57, 67, 69, 70 and 80 years-old), which were cultured in an osteogenic-inducing medium in the absence or presence of mechanical stimulation. The cells from 4 out of 6 Pm women were also incubated with A438079 (3 µM, a selective P2X7 receptor antagonist) and MRS 2578 (0.1 µM, a selective P2Y6 receptor antagonist). The house keeping gene protein product, β-Tubulin (55 kDa), was used for normalization purpose. Dashed boxes indicate cropped regions of the blots depicted in Fig. 6 (panels A and B). [file 13287_2024_3775_MOESM2_ESM.tif]

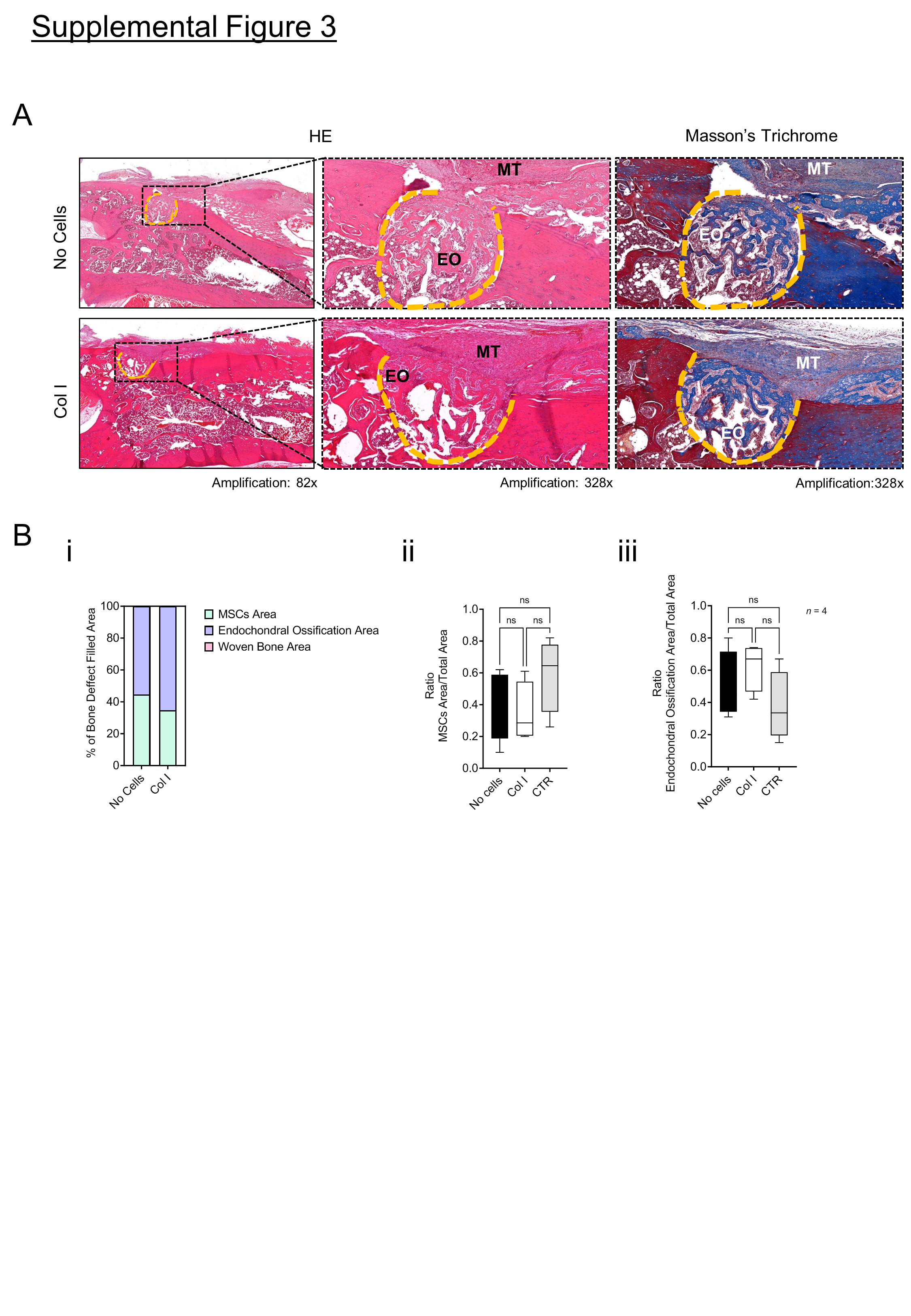

Supplement: Supplementary file 3 — Supplementary Material 3: Suppl Fig. 3. Sham (no-cells) and collagen I (Col I)-loaded femoral defects used as controls for the in vivo experiments. Panel A shows representative micrograph sections of femoral defects stained with hematoxylin and eosin (HE) and Masson’s trichrome at different magnifications (82x and 328x) taken from sham (no-cells) and collagen I (Col I)-loaded defects used as controls of data shown in Fig. 7. Bone defects are outlined with a dashed yellow line. MT represents the mesenchymal tissue comprising autologous MSCs recruited to the injury site; EO denotes endochondral ossification as result of secondary bone repair. Panel B shows three graphs computed from histological digital images representing (i) the percentage of MSCs area, endochondral ossification area and woven bone area as a function of the bone defect-filled area (100%); (ii) the ratio between MSCs and bone defect total area; and (iii) the ratio between endochondral ossification and bone defect total area, for each experimental condition. Data obtained with transplanted BM-MSCs differentiated for 21 days in an osteoblast-inducing medium (CTR), is also shown for comparison. Boxes and whiskers represent pooled data from 4 individual experiments; the cells were obtained from the same 4 Pm women (60, 68, 76 and 79 years old) used in Fig. 7. Not significant (ns; ordinary one-way ANOVA with Fisher’s LSD test). [file 13287_2024_3775_MOESM3_ESM.tif]
